# Combinatorial engineering of betalain biosynthesis pathway in yeast *Saccharomyces cerevisiae*

**DOI:** 10.1186/s13068-023-02374-4

**Published:** 2023-08-17

**Authors:** Mahsa Babaei, Philip Tinggaard Thomsen, Jane Dannow Dyekjær, Christiane Ursula Glitz, Marc Cernuda Pastor, Peter Gockel, Johann Dietmar Körner, Daniela Rago, Irina Borodina

**Affiliations:** The Novo Nordisk Foundation Center for Biosustainability, Technical University of Denmark, Kemitorvet Building 220, 2800 Kgs. Lyngby, Denmark

**Keywords:** Betanin, Betaxanthins, Betalains, Metabolic engineering, *Saccharomyces cerevisiae*

## Abstract

**Background:**

Betalains, comprising red–violet betacyanins and yellow–orange betaxanthins, are the hydrophilic vacuolar pigments that provide bright coloration to roots, fruits, and flowers of plants of the Caryophyllales order. Betanin extracted from red beets is permitted *quantum satis* as a natural red food colorant (E162). Due to antioxidant activity, betanin has potential health benefits.

**Results:**

We applied combinatorial engineering to find the optimal combination of a dozen tyrosine hydroxylase (TyH) and 4,5-dopa-estradiol-dioxygenase (DOD) variants. The best-engineered *Saccharomyces cerevisiae* strains produced over six-fold higher betaxanthins than previously reported. By genome-resequencing of these strains, we found out that two copies of DOD enzyme from *Bougainvillea glabra* together with TyH enzymes from *Abronia nealleyi*, *Acleisanthes obtusa*, and *Cleretum bellidiforme* were present in the three high-betaxanthin-producing isolates. Next, we expressed four variants of glucosyltransferases from *Beta vulgaris* for betanin biosynthesis. The highest titer of betanin (30.8 ± 0.14 mg/L after 48 h from 20 g/L glucose) was obtained when completing the biosynthesis pathway with UGT73A36 glucosyltransferase from *Beta vulgaris*. Finally, we investigated betalain transport in CEN.PK and S288C strains of *Saccharomyces cerevisiae* and identified a possible role of transporter genes *QDR2* and *APL1* in betanin transport.

**Conclusions:**

This study shows the potential of combinatorial engineering of yeast cell factories for the biotechnological production of betanin.

**Supplementary Information:**

The online version contains supplementary material available at 10.1186/s13068-023-02374-4.

## Background

Color is an essential characteristic of food, associated with quality, freshness, and taste perception. After the industrial revolution and increased consumption of processed foods, natural and synthetic food dyes have become important tools in food industries to enhance or correct color variations and give an expected color to otherwise colorless food [1]. The regulation of food colors differs between countries, for example, only six synthetic food colors are approved in both the US and Europe [2]. In the last decade, especially after the publication of the Southampton study [2–5], the growing customer awareness of the possible harmful effects of synthetic colors has made the food industry move away from synthetic colors toward natural colorants. Studies have indicated that synthetic food colors may trigger hypersensitivity reactions, cancer in animal studies [6–8], and hyperactivity in children [3].

Betalains are a group of water-soluble, natural pigments that are divided into the yellow–orange betaxanthins (absorption maxima *λ*_max_ = 475 nm) and the red–violet betacyanins (*λ*_max_ = 540 nm) [9]. Betalains can be obtained by extraction from red beets and prickly pear cactus. The major color component in red beet is betanin, while it is a mixture of indicaxanthin and betanin in preickly per cactus [10, 11]. Compared to other natural colors such as anthocyanins, betalains possess higher (up to three times) tinctorial strength and are stable in a relatively wider pH range of 3 to 7 [12]. Furthermore, betalains have an extraordinarily strong antioxidant capacity, as these compounds can decrease oxidative stress by efficiently removing reactive oxygen species [13, 14]. Multiple in vivo studies support the biological activity of these pigments [15–19]. In humans, daily uptake of 100 mg red beet concentrate (with a minimum content of 25% wt betalain) promoted an anti-inflammatory response [20]. So far, betalains have been found in the Caryophyllales order of plants [21], in the fungi *Amanita* [22], and *Hygrocybe* [23], and recently in the bacteria *Gluconacetobacter diazotrophicus* [24] and cyanobacteria *Anabaena cylindrica* [25]. Among the Caryophyllales plants, the red beet *Beta vulgaris*, prickly pear cactus *Opuntia ficus-indica*, garden four-o'clock *Mirabilis jalapa*, paper flower *Bougainvillea glabra*, and *Portulaca grandiflora* are examples of betalain producing plants [26]. As of 2015, the structures of approximately 75 distinct betalains have been identified from 17 different plant families [26]. With the development of new analytical methods in liquid chromatography and mass spectrometry, the number of identified betalains is continuously increasing [27].

**Fig. 1:**
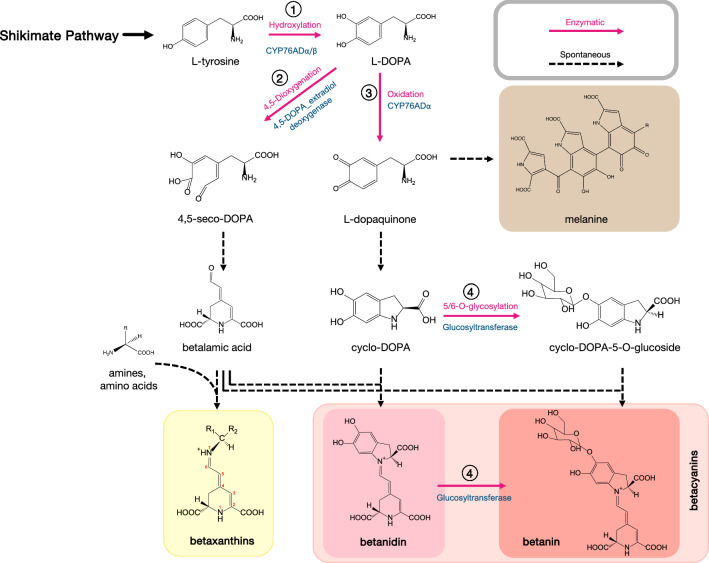
Biosynthetic pathway from *L*-tyrosine towards betalains

The biosynthesis of betalains (Fig. 1) starts with hydroxylation of *L*-tyrosine to L-DOPA (L-3,4 dihydroxyphenylalanine), catalyzed by cytochrome P450 monooxygenase CYP76AD. By the action of an extradiol dioxygenase enzyme (DOD), the cyclic ring of L-DOPA is opened between carbons 4 and 5, forming the unstable 4,5-*seco*-DOPA. This compound is then non-enzymatically rearranged to betalamic acid, the basic chromophore in all betalains. The color of betalains is related to the formation of a conjugated π-electron system of the 1,7-diazaheptamethin that is created by Schiff-base condensation of a nucleophilic amine and betalamic acid aldehyde group [26]. L-DOPA is also the precursor of *cyclo*-DOPA that is synthesized by a sub-clade of the enzyme family, CYP76AD [28].

Commecial betalains are made by extracting shredded raw beetroots with acidic water solution. Betanin content in red beets is low (0.03–1.3%) [29, 30], and the extract contains high concentrations of sugars and nitrates, which necessitates further processing steps, such as fermentation and reverse osmosis [31]. Furthermore, beetroots contain some geosmin and various pyrazines [32], which give an earthy smell to the beetroot extracts and limits the maximum applicable concentrations in some foods, such as dairy products [33]. Also, due to the presence of endogenous enzymes such as β-glucosidases, peroxidases, and polyphenoloxidases in red beet extract, the betalains can easily degrade in the beetroot juice if not blanched [34]. With advances in synthetic biology, the production of betalains by microbial fermentation can become an alternative to extraction from plants. To this end, Hou et al. have engineered *Escherichia coli* to produce betalamic acid by overexpressing the endogenous tyrosine hydroxylase (HpaBC) together with an extradiol dioxygenase from *M. jalapa* (*Mj*DOD) [35]. By feeding different amino acids and amines to the betalamic acid-producing strain BTA6, they could obtain betaxanthins and betacyanins in the presence of ascorbic acid as the antioxidant agent. However, feeding amino acids and amines and ascorbic acid at large-scale fermentation would result in high production costs. With baker's yeast as the host strain, Grewal et al. [36] reported production of 17 mg/L betanin in strain yCM363 (BY4741, *leu2∆*:: *Mj*cDOPA5GT, *ura3∆*:: *Mj*DOD-*Bv*CYP76AD1^W13L^-*Sc*ARO4^K229L^) from 20 g/L glucose in minimal media without any antioxidant supplemented. The W13L point mutation in the enzyme *Bv*CYP76AD1^W13L^ was reported by the same group to give a 2.8-fold increase in betanidin titer compared to the wild type enzyme variant [37]. Recently, Zhang et al. reported the highest titer of 28.7 mg/L in 72 h from 20 g/L glucose in minimal medium through fermentation optimization and pathway-enzyme modification in yeast *S. cerevisiae* [38].

In the metabolic engineering of heterologous plant pathways, selecting appropriate genetic sources for enzymes is a challenging task, mainly due to the unpredictable expression and activity of these enzymes within non-native hosts [39] and inaccurate function annotations in plant genomes [40]. According to Gandia-Herrero et al. there are 31 glucosyltransferase sequences from betalain-producing plants deposited but only a few are characterized as active towards the substrates in the pathway [41]. This study aims to identify a suitable combination of betanin biosynthetic enzymes for yeast cell factories through a systematic combinatorial approach. To accomplish this, we implemented a high-throughput engineering method and coupled it with the FACS technique, leveraging the fluorescence of betaxanthins to identify superior enzyme combinations. Further, we explored multiple glucosyltransferase variants from the red beet and identified two active ones.

## Results

### Combinatorial assembly of betaxanthin pathway in yeast ***S. cerevisiae***

**Fig. 2:**
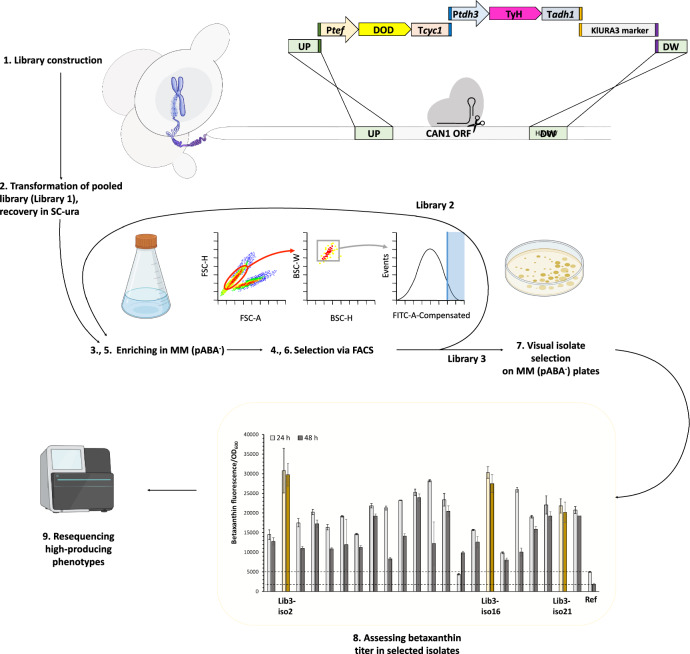
The scheme of combinatorial engineering of yeast for improved production of betaxanthins. A combinatorial library of DOD and TyH enzymes was assembled in yeast and the population was enriched for high fluorescence over two rounds of FACS and plated. Individual colonies were cultivated in liquid medium in deep-well plates and fluorescence was measured. The “Ref” strain harbors integration of *Bv*CYP76AD^W13L^ and *Mj*DOD in the same strain background as library isolates. The three highest fluorescent isolates from Library 3 (iso2, iso16 and iso21) were genome sequenced. Data shown are mean values ± SDs of biological triplicates

To obtain yeast strains with high betaxanthin production, a combinatorial library of genes encoding the two proteins of the biosynthetic pathway, *i.e.,* tyrosine hydroxylase (herein named TyH) and 4,5-dopa-extradiol-dioxygenase (DOD), was integrated into the genome of *S. cerevisiae* using the method described by Kildegaard et al. 2019 [42]. The schematic overview of the method is shown in Fig. 2, with the list of gene orthologs in Table 2. In summary, the parent strain ST8251 (CEN.PK113-5D expressing *cas9* gene) was transformed with a gRNA plasmid targeting the *CAN1* locus (pCfB2310, Additional file 1: Table S4), and five elements of overexpression cassettes for in vivo assembly. The five parts of the overexpression cassettes consisted of: (i) the upstream homology arm for the *CAN1* site (BB0629, Additional file 1: Table S2) (ii) twelve DOD variants under the control of *TEF1* promoter (P*tef1*) and *CYC1* terminator (T*cyc1*), (BB4733:BB4743, Additional file 1: Table S2), (iii) eleven TyH variants under the control of *TDH3* promoter (P*tdh3*) and *ADH1* terminator (T*adh1*), (BB4744:BB4753, Additional file 1: Table S2), (iv) auxotrophic marker Klura3 from *Kluyveromyces lactis* (BB4732, Additional file 1: Table S2), and (v) the downstream homology arm for *CAN1* site (BB0630, Additional file 1: Table S2).

To benchmark the performance of our library of gene orthologs, we used the most efficient gene variants reported so far in the literature, which were the mutated version of *B. vulgaris* TyH (*Bv*CYP76AD^W13L^) together with *M. jalapa* DOD (*Mj*DOD) [37]. The *S. cerevisiae* codon-optimized versions of these two genes were expressed in the same strain background and with the same method as the library, giving the reference strain ST10319. After the yeast library construction, the fluorescence signal of the cell population was evaluated against the negative control (parent strain, ST8251) and the positive control (ref strain, ST10319) to sort for single cells with a higher fluorescence signal than in the reference strain (ST10319, Additional file 1: Fig. S1). After two rounds of sorting, the average fluorescence values for Library 3 population increased by an order of magnitude (Mean = 5660, Median = 5097) compared to the reference strain (ST10319: Mean = 455, Median = 405).

We then proceeded with cultivating Library 3 on modified mineral medium MM (pABA^−^) plates, and visually selected 22 single isolates with intense yellow color and measured the fluorescence/OD_600_ ratio after 24 and 48 h of cultivation. For most isolates, there was higher betaxanthins production compared to the reference strain ST10319 (step 8 in Fig. 2). The reason behind slightly higher level of betaxanthins titer at 24 h compared to 48 h is due to the instability of betaxanthin molecules, which are described to degrade and loss in color [43]. To verify the origin and the copy number of the genes providing the desired phenotype, we performed whole-genome re-sequencing of the top three betaxanthin producers in Fig. 2, i.e., iso2, iso16, and iso21. The presence and identity of the integrated variants was experimentally verified by obtaining a PCR product for each gene using specific primer pairs and the isolated genomic DNA as the PCR template. These fragments were also analyzed by Sanger sequencing (Eurofins Genomics, Ebersberg, Germany). Genome resequencing showed the presence of three copies of TyH and DOD genes in each isolate (Table 1). Besides the intended integration of the genes to CAN1 locus on ChrV, for all three isolates we located the extra two copies of DOD genes in TEF1 and CYC1 loci, on ChrXVI and ChrX, respectively. In similar way, the extra two copies of TyH genes were integrated into TDH3 and ADH1 loci, on Chr VII and ChrXV, respectively. The homology of transcriptional elements (promoters P*tef1* and P*tdh3*, and terminators T*cyc1* and T*adh1*) present in overexpression cassettes and *S. cerevisiae* genome is probably the main reason for multi-integration of pathway genes. Furthermore, in all the three isolates, we observed the integration of DOD gene from *B. glabra (Bg*DOD1) in two copies together with a single copy of thymine-732 deleted DOD gene from *P. grandiflora* (*Pg*DOD^*T732∆*^). The TyH genes from *C. bellidiforme* was common in all the enriched isolates. Since the monooxygenase activity of single *An*TyH, *Ao*TyH and *Cb*TyH is previously described to be low for norcoclaurine (a dopamine derivative) biosynthesis in yeast [44], the integration of multiple TyH genes enabled high betaxanthin production in the selected top library isolates.

**Table 1:** Integrated DOD and TyH genes in the genome of top betaxanthin producers according to sequencing data

**Isolate**	ST8251	iso2	iso16	iso21
Integrated DODs	–	*Bg*DOD1 (2x) *Pg*DOD^*T732∆*^	*Bg*DOD1 (2x) *Pg*DOD^*T732∆*^	*Bg*DOD1 (2x) PgDOD^*T732∆*^
Integrated TyHs	–	*An*TyH *Ba*TyH *Cb*TyH	*Ao*TyH *Ba*TyH *Cb*TyH	*An*TyH (2x) *Cb*TyH

**Fig. 3:**
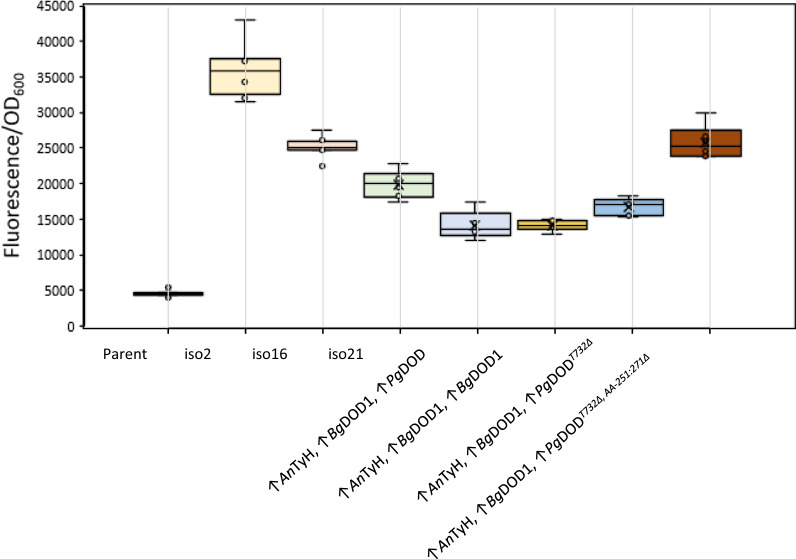
Investigating the possible synergistic interaction between T732-deleted *Pg*DOD and *Bg*DOD1 on betaxanthin fluorescence. The integration of intact *Pg*DOD or *Bg*DOD1 alongside *An*TyH-*Bg*DOD1 did not result in any betaxanthin titer close to library isolates. However, by expressing *Pg*DOD^*T732∆*^ an increase in fluorescence is observed, and by expressing the truncated version *Pg*DOD^*T732∆, AA−251:271∆*^ (deleted tail after the premature stop codon at AA_251_) the betaxanthin content was restored to that of Lib3-iso16. Data shown are mean values ± SDs of *n* = 6 biological replicates

To understand the reason behind the enrichment of thymine-732 deleted DOD gene from *P. grandiflora* (*Pg*DOD^*T732∆*^) in all the top-producing isolates, we tried integrating this mutated version together with two different TyH genes, *An*TyH and *Ev*TyH. However, none of the obtained variants showed the yellow phenotype for betaxanthins production (Additional file 1: Fig. S2). The in silico analysis showed that *T*_*732*_*Δ* mutation results in a frameshift in AA_242_ (AA: amino acid) of *Pg*DOD protein and introduces a premature stop codon in AA_251_ of mutated protein (Additional file 1: Table S1). Moreover, identification of DOD from *B. glabra (Bg*DOD1) along with *Pg*DOD^*T732∆*^ in all three isolates might imply a synergistic interaction between these two specific DOD gene orthologs. To further investigate this, we constructed different strains by integrating single copies of *Bg*DOD1 and *An*TyH, as the latter was one of TyH orthologs commonly found in the three enriched isolates (Table 1). We then investigated any potential epistatic interaction between the mentioned DOD gene orthologs by adding mutated *Pg*DOD^*T732∆*^ and the truncated version of this protein (*Pg*DOD^*T732∆, AA−251:271∆*^) where the tail after the premature stop codon in AA_251_ was removed. As controls, we also constructed two strains with intact *Pg*DOD and *Bg*DOD1, to decipher the effect of increasing DOD gene copy number from synergistic interactions between the enzyme orthologs. After cultivating these strains together with Lib3 isolates iso2, iso16 and iso21 for betaxanthin production, we observed a significant (app. twofold) increase in titers for the strain with *Bg*DOD1 and *Pg*DOD^*T732∆, AA−251:271∆*^ compared to intact *Pg*DOD or *Bg*DOD1 (Fig. 3).

### Identification of UGTs from *B. vulgaris* for betanin production in yeast

To obtain a yeast strain producing betacyanins, we moved forward to search for novel glucosyltransferases in the genome of *B. vulgaris*. We used the protein sequence of betanidin/cyclo-DOPA glucosyltransferase from *Beta vulgaris* (GenBank: AAO88911.1) as the query. This gene was BLASTed against the RefBeet-1.2.2 *Beta vulgaris* genome (Bioproject PRJNA41497 [45–47], and for each of the BLAST hits, five coding DNA sequences located up- and downstream (5’- and 3’-ends, respectively) of the BLAST hits were inspected. Of these genes, four CDSs with glucosyltransferase in the annotation were identified, all of them annotated as "scopoletin glucosyltransferase". These genes were UGT73DN1, UGT73A36, UGT73A38, and UGT73A39, with their protein sequences and corresponding codon-optimized sequences for *S. cerevisiae* provided in Additional file 1: Table S1. To benchmark the activity of these UGTs, we used the betanidin-5-O- glucosyltransferase from *D. bellidiformis* (*Db*B5GT), since this enzyme was reported to have a high efficiency toward betanin production [36].

**Fig. 4:**
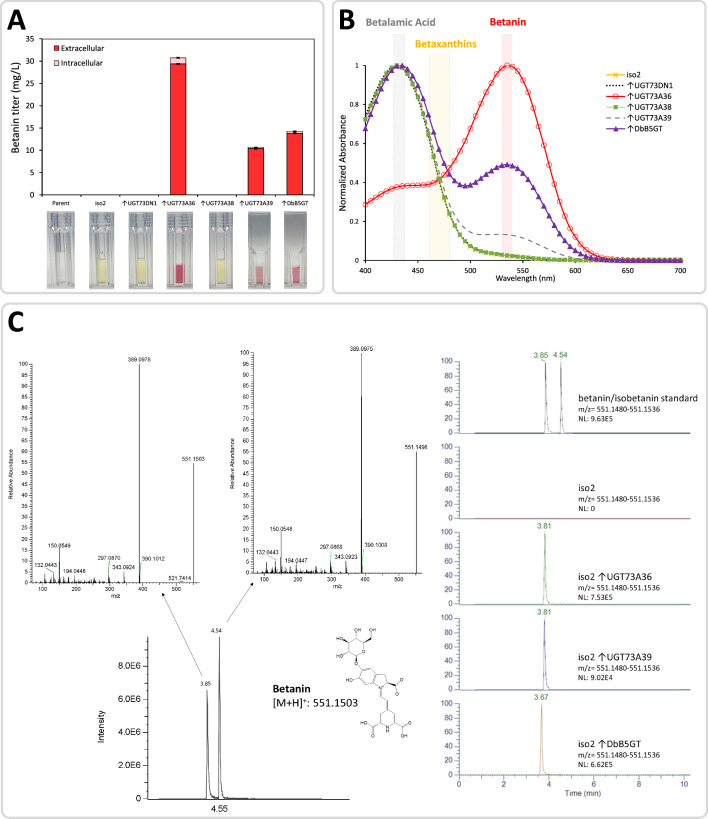
Betanin production in yeast strains expressing UGTs from *B. vulgaris*. **A** Betanin titer (mg/L) measured as extracellular and intracellular content after 48 h of cultivation in MM (pABA^−^) media supplemented with 20 g/L glucose. The color of culture supernatant is also shown for the strains. **B** Normalized UV–visible spectra of the extracted cultures. Betanin at maximum absorbance of 534 nm is present in strains with integrated UGT73A36, UGT73A39, and *Db*B5GT. **C** Mass spectrometry analysis of betacyanins. The presence of isobetanin (retention time at 4.54 min) and betanin (retention time 3.85 min) in beetroot extract standard sample is shown in mass spectra **(left)**. The spectra of the yeast cultures **(right)** confirm the synthesis of betanin (and not isobetanin) in strains with integrated UGT73A36, UGT73A39, and *Db*B5GT

To assess the activity of identified UGT variants, we first transformed the betaxanthin producing isolate iso2 with pCfB2312 for *cas9* expression, as this plasmid was lost in the library cells due to the lack of G418 antibiotic selection during propagation and sorting. We then constructed five strains by transforming four UGTs alongside *Db*B5GT into iso2 expressing Cas9 protein. After small-scale cultivation of these strains for 48 h, we observed a vivid red color in the strains expressing UGT73A36, and a pink color in strains with UGT73A39 (Additional file 1: Fig. S3 and Fig. 4A). The synthesis of betanin was quantified by HPLC (Fig. 4A), with the highest titer of 30.8 ± 0.14 mg/L in strain expressing UGT73A36 after 48 h growth in minimal medium containing 20 g/L glucose. Similarly, integration of UGT73A39 resulted in 10.7 ± 0.03 mg/L of betanin synthesis (Fig. 4A). The synthesis of betanin was also confirmed by UV–visible spectra of the culture broth (Fig. 4B) which showed the bathochromic shift in absorption maxima from 475 nm (betaxanthins) to 535 nm for betacyanins. The mass spectrometry analysis (Fig. 4C) also confirmed the presence of betanin in fermentation broth of strains expressing UGT73A36, UGT73A39 and DbB5GT, compared to betanin standard from beetroot extract. It is worth mentioning that isobetanin was not detected in any of the engineered strains.

### *In-vitro* glycosylation of UGT73A36

**Fig. 5:**
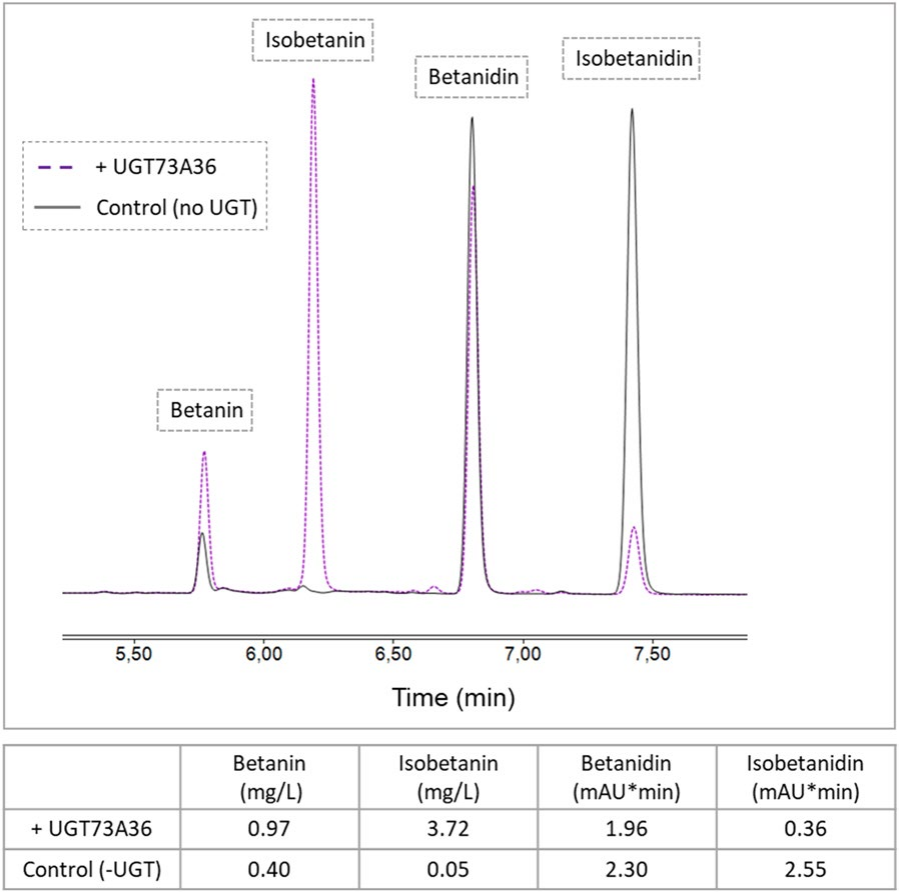
HPLC analysis of *in-vitro* glycosylation assay of UGT73A36. Chromatograms show the reaction products when 20 µM betanidin were mixed with 5 µL of purified UGT73A36 (dashed line) or with water (solid line). The table illustrates the amount of (iso-) betanin and (iso-)betanidin present in the mix after the reactions with or without purified enzyme

To analyze the in vitro glycosylation activity of the in vivo best performing glycosyltransferase, UGT73A36, the enzyme was recombinantly expressed in *E. coli* BL21 and purified by affinity chromatography. SDS-PAGE analysis revealed a high purity of the purified protein and a concentration of 10.3 mg/mL was determined by BCA assay (Additional file 1: Fig. S6). The *in-vitro* reaction with 20 µM betanidin as substrate showed a regioselective transfer of the glucose moiety to the 5-hydroxylgroup of betanidin or isobetanidin, resulting in the formation of betanin and isobetanin, respectively. While most of the isobetanidin was glycosylated to isobetanin (86%), only 15% of the betanidin was glycosylated to betanin (Fig. 5).

### Investigating betalain transporters

From a manufacturing point of view, it is of enormous advantage if the engineered cell factories are efficient in secretion of final products (in this case betanin) while retaining the pathway intermediates (betalamic acid and betaxanthins) [48]. Looking at Fig. 4A, we can see that our engineered strains are effective in secreting betanin into culture medium, with 95.6% of total betanin content being secreted to the medium in our best performing strain (iso2 expressing UGT73A36). However, our results are different from what was reported by Grewal et al. where only two-third of the synthesized betanin was secreted into the cultivation medium [36]. Since the analytical methodology and sample preparation was identical in both studies, we speculated that the genetic background of the strains, CEN.PK strain in our study versus S288C in the study by Grewal et al. [36]., might explain this discrepancy.

**Fig. 6:**
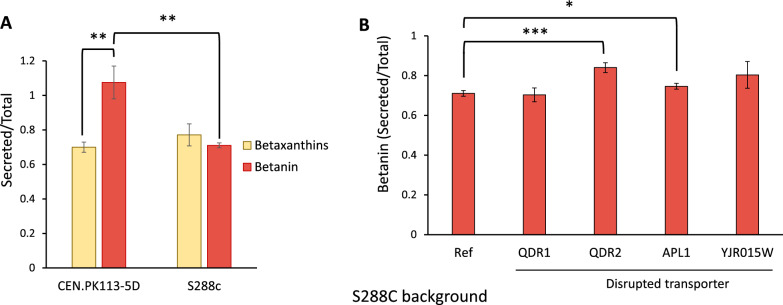
**A** The difference in secretion of betalains in strains derived from CEN.PK and S288C backgrounds. Betaxanthins were measured in strains of CEN.PK113-5D and S288C with integrated *Mj*DOD and *Bv*CYP76AD^W13L^, and betanin was measured for similar strains with additional UGT73A36 integrated. Betalain export is highly dependent on the strain background. **B** Effect of disruption of transporter genes on secreted fraction of betanin in S288C background. Data shown are mean values ± SDs of biological triplicates. Statistical difference was determined by one-tailed, two-sample unequal variance; *p < 0.05; **p < 0.01; ***p = 0.001

To elaborate on this, we examined the effect of strain background on betalains (betanin and betaxanthins) secretion. By integrating similar pathway genes in strains CEN.PK113-5D and S288C (Fig. 6A) we noticed that S288C-derived strains retrained about 30% of betanin inside the cells when compared to CEN.PK which had 100% of betanin secreted to the culture broth (*p*-value < 0.01). We could also observe a difference in metabolite secretion capacity in the same strain *i.e.*, CEN.PK is more efficient in betanin than betaxanthin export (*p*-value < 0.01). In case of S288C, this strain showed equal performance in exporting betaxanthins and betanin (*p*-value > 0.1), which would act as a disadvantage in developing a cell factory, since the former serves as the precursors of betanin. Furthermore, to investigate whether we could enhance the secretion of betanin in S288C background, we applied findings from our previous study on high-throughput screening of transporter mutants for betaxanthin secretion in yeast [49]. Using a CRISPR-Cas9 mediated method for the disruption of native transporters in *S. cerevisiae*, four transporters related to betaxanthins were identified in that study: quinidine/cations-related transporters *QDR1* and *QDR2*, a subunit of the clathrin-associated protein complex *APL1*, and a putative transporter *YJR015W*. We then used the same method to disrupt each of these genes in a S288C-derived betanin producing strain (Fig. 6B) by introducing a frameshift in the ORF regions leading to premature translation termination. We observed that disruption of *QDR2* and *APL1* genes was associated with improved betanin secretion of 18.3% and 5.0% compared to the Ref strain, respectively.

## Discussion

As an alternative to plant-extracted betalains, production of these pigments in microbial cell factories allows for synthesis of individual colorants with higher productivity and consistent quality. However, the selection of enzyme candidates for assembly of plant pathways in recombinant hosts is challenging. To the authors’ knowledge, there is no concise study on the comparison of enzymatic activity in the betalain biosynthesis pathway, namely 4,5-DOPA-extradiol dioxygenases (DOD) and cytochrome P450 monooxygenase CYP76ADα (TyH) in combination.

Our first aim in this study was to screen a large set of DOD and TyH gene orthologs from different betalain- producing plants in a combinatorial way to obtain strains with high betaxanthin production. To this end, we enriched for multiple yeast isolates with improved betaxanthin production compared to a benchmark strain (expressing *Bv*CYP76AD^W13L^ and *Mj*DOD [37]). Genome re-sequencing of the three highest-betaxanthin producing isolates showed integration of *Bg*DOD1 from *B. glabra* in all isolates in more than one copy. For this enzyme, Sasaki et el. have reported that they observed enzymatic activity of purified protein, but no data on production titers were provided [50]. Furthermore, according to the study by Brockington et al. [51] the presence of amino acid residues Pro-183 and Trp-227 in *Bg*DOD1 and *Pg*DOD would place these two proteins in clade DODα, which have higher activity in betalain synthesis than β-clade (with Asn-183 and Ala-227). The enrichment of thymine-732 deleted DOD enzyme *Pg*DOD^*T732∆*^ together with *Bg*DOD1 in all the three isolates pointed to a possible synergistic effect of *Bg*DOD1-*Pg*DOD^*T732∆*^ on betaxanthin titer improvement, an effect which was further pronounced when a truncated *Pg*DOD version, with the deleted tail after the premature stop codon in AA_251_, was overexpressed. The identification of dimer forms of 4,5-dopa-extradiol dioxygenase enzymes in bacteria *G. diazotrophicus* [24] and *A. cylindrica* [25] gives rise to the hypothesis that *Bg*DOD1-tr*Pg*DOD^*T732∆*^ might form an in vivo protein dimer, which improves the enzymatic activity of these proteins. However, this hypothesis needs to be further analyzed and validated for purified proteins. To address the issue on multi-integration of pathway genes which is due to efficient homologeous recombination mechanism in *S. cerevisiae*, we suggest to use synthetic or hybrid-synthetic promoters and terminators [52–54] to construct the orthogonal genetic components for yeast.

One of the major issues in the production and application of betalains is the stability of these pigments. In the plant kingdom, the route to stabilize the betacyanin backbone molecule, *i.e,.* betanidin, is the O-glycosylation at positions C_5_ or C_6_ of cyclo-DOPA, giving betanin or gomphrenin I, repectively. Betanin (betanidin 5-O-β-glucoside) shows 17 times higher half-life value compared to betanidin due to higher oxidation–reduction potential of glycosylated form [55]. The UGT enzymes screened in this study UGT73A36 and UGT73A39 with accession no. KMT01176.1 and KMT01177.1, respectively, were efficient enzymes that showed high in vivo activity in betanin production. During the process of drafting this manuscript, we found out about the flavonoid-specific glucosyltransferase UGT73A4 identified by Isayenkova et al. from *B. vulgaris* genome in 2006 [56] that was characterized to catalyze betanidin 5-O glycosylation in vitro. Despite 99% identity between this protein and UGT73A36 from our study, this protein (locus: chromosome 9, 34412950:34414381) has not been annotated accordingly in reference beetroot genome, RefBeet 1.2.2 in 2015^1^ (Bioproject PRJNA41497 [45–47]. Besides this, in their study, Isayenkova et al. showed that the catalytic efficiency for the protein UGT73A4 towards betanidin is far less than flavonoids, which they state that “*makes its *in vivo* role in betanin biosynthesis questionable*” [56]. We showed that this protein (with UGT nomenclature of UGT73A36 here) is in fact active in vivo for betanin synthesis. Characterization of the in vitro glycosylation activity of UGT73A36 showed that the purified enzyme is active and takes betanidin and isobetanidin as substrate. While Isayenkova et al. determined almost no activity of purified UGT73A36 on betanidin (efficiency of 0.04%, [56]), we could show that under the given conditions, 85% of the isobetanidin and 15% of the betanidin were glycosylated by the enzyme to isobetanin and betanin, respectively. This suggests a higher substrate specificity of UGT73A36 for isobetanidin than for betanidin. To further characterize the enzyme, kinetic analyses with betanidin as well as with other substrates could be performed but the relevance of these results for in vivo production of betanin in is limited.

One of the potential advantages of betalain production in microbial hosts is their ability to export the end product, which simplifies the downstream purification especially when considering the traditional maceration and extraction process of betanin from beetroot [31]. We found that secretion of betanin in *S. cerevisiae* is highly dependent on strain background, with the CEN.PK strain being more efficient in betanin export compared to the S288C strain, likely due to differences in the native transporters [49] in the two strains. Furthermore, we observed that deletion of *QDR2* and *APL1* transporters positively affected betanin secretion in S288C strain. In case of *QDR2*, the null mutant retained a higher fraction of betaxanthins inside the cells [57], which would increase the intracellular content of betanin precursors.

Overall, this study is focused on two main areas required for developing a cell factory of *S. cerevisiae* for betanin biosynthesis: pathway optimization through screening of key enzymes from different plant origins and investigating the role of some of indigenous transporters for final product secretion to the culture broth. The results of this study are promising in terms of establishing a versatile betalain synthesis platform in *S. cerevisiae* that would enable future optimization for industrial production.

## Conclusions

Combinatorial reconstruction of a multi-enzyme pathway allowed rapid identification of enzyme combinations beneficial for the production of betaxanthins in yeast. Supplementation of the pathway with glucosyltransferase UGT73A36 or UGT73A39 from the red beet *Beta vulgaris* enabled biosynthesis of betanin, one of the most used natural red food colors. Considerable further strain engineering will be necessary for commercial-scale fermentative production of betanin, but such engineering can also be performed in a combinatorial manner, reducing the time from research to the market.

## Methods

### Synthetic genes and DNA materials

The heterologous genes (Table 2, Additional file 1: Table S1) were all synthesized by GeneArt (Life Technologies) in codon-optimized versions for *S. cerevisiae*. All DNA parts were PCR amplified using Phusion U DNA polymerase (ThermoFisher) according to the manufacturer's instructions. The DNA fragments (BioBricks) are listed in Additional file 1: Table S2, with primer sequences listed in Additional file 1: Table S3. The DNA fragments obtained by PCR were separated in 1%-agarose containing RedSafe™ (iNtRON Biotechnology) and purified using the Nucleospin Gel and PCR Clean-up kit (Macherey–Nagel). Integrative vectors (Additional file 1: Table S4) were constructed as described in EasyClone MarkerFree method [58].

**Table 2:** List of enzyme variants

Variant	Nomenclature	Organism	GenBank
4,5-dopa-extradiol dioxygenase (DOD)	*Mj*DOD	*Mirabilis jalapa*	B6F0W8.1
	*Bv*DOD1	*B. vulgaris*	Q70FG7.1
	*Bg*DOD1	*Bougainvillea glabra*	ASW22755.1
	*Pg*DOD	*Portulaca grandiflora*	CAE45178.1
	*So*DOD	*Spinacia oleracea (spinach)*	XP_021836119.1
	*At*DOD	*Amaranthus tricolor*	AJW81119.1
	*Ah*DOD	*Amaranthus hypochondriacus* (grain amaranth)	ADZ48644.1
	*Pa*DOD	*Phytolacca americana* (American pokeweed)	BAH66635.1
	*Ss*DOD	*Suaeda salsa*	ACO59903.1
	*Bg*DOD2	*B. glabra*	BAG80687.1
	*Bv*DOD2	*B. vulgaris*	AET43293.1
	*Bv*DOD3	*B. vulgaris*	AET43287.1
Tyrosine hydroxylase (TyH)	*BvCYP76AD*^*W13L*^	*B. vulgaris*	AET43289.1
	*Ba*TyH	*Basella alba*	AJD87470.1
	*Cb*TyH	*Cleretum bellidiforme*	AJD87468.1
	*Pa*TyH	*Phytolacca americana*	AJD87467.1
	*Of*TyH	*Opuntia ficus-indica*	AJD87464.1
	*An*TyH	*Abronia nealleyi*	AKI33952.1
	*Ao*TyH	*Acleisanthes obtusa*	AKI33950.1
	*Mm*TyH1	*Mirabilis multiflora*	AKI33948.1
	*Ev*TyH	*Ercilla volubilis*	AKI33945.1
	*Pd*TyH	*Phytolacca dioica*	AKI33942.1
	*Mm*TyH2	*Mirabilis multiflora*	AKI33937.1

### Screening orthologous genes for DOD and CYP76ADα (TyH)

To find enzymes for 4,5-dopa-extradiol-dioxygenase (DOD), 229 plant protein sequences were selected from 814 available sequences in NCBI using the search term DOPA. The sequences were filtered by annotation to remove sequences that were partial, putative, hypothetical, uncharacterized, or predicted-"like". Sequences not containing the term "DOPA" were also removed. The resulting 65 sequences were screened based on a multiple sequence alignment to exclude sequences shorter than 260 amino acids and longer than 310 amino acids. The sequences were analyzed for; (1) the absence of conserved motifs for DOD from non-betalain producing plants His-177 followed by N-L-(R/G) [59], and then (2) the presence of the conserved pattern for DOD proteins from betalain-producing plants, H-P-(S/A)-(N/D)-x-T-P motif [59]. The final list was checked for similarity by MATGAT (Additional file 1: Fig. S4) [60]. Final screened variants of DOD proteins are listed in Table 2, with the codon-optimized sequences for *S. cerevisiae* detailed in Additional file 1: Table S1.

Similarly, for CYP76ADα (termed as TyH in this study) 35 sequences of plant CYP76AD1, complete and annotated as P450 or CYP, were selected from the NCBI database based on a search for the term CYP76AD1 in plants. The sequences were screened for the presence of P450 signature motifs (A/G)-G-x-(E/D)-T-(T/S), E-x-x-R (KETLR-motif), P-(E)-R-(F) (PERF-signature), and the heme-binding motif F-x-x-G-x-R-x-C-x-G [61]. The filtered sequences were additionally checked for the presence/absence of amino acids that characterize 76AD1 family clade α as described by Sunnadeniya et el. [28]. The remaining 14 sequences were checked for similarity to ensure that none of the sequences were identical, using MATGAT (Additional file 1: Fig. S5) [60]. Final screened variants of TyH proteins are listed in Table 2, with the *S. cerevisiae* codon-optimized sequences in Additional file 1: Table S1.

### Strains and media

To clone and store plasmids (Additional file 1: Table S4), *E. coli* strain DH5α was used. The cultivations were carried out at 37°C in Lysogeny Broth (LB) broth or on agar plates supplemented with 100 mg/L ampicillin as selection marker. The yeast strain CEN.PK113-5D (MATa ura3-52 HIS3 LEU2 TRP1 MAL2-8c SUC2) and BY4741 (MATa HIS3Δ1 LEU2Δ0 MET15Δ0 URA3Δ0) both harboring episomal vector for Cas9 protein expression (P*tef1*-Cas9-T*cyc1*_kanMX) were used as the parent strains (ST8251 and ST9771, respectively) in this study for constructing all *strains* (via rational genome engineering), or *isolates* (via high-throughput genome modification) [62]. To keep the selection for Cas9, the cultivations were supplemented with 200 mg/L G418 (Sigma–Aldrich). The construction of betacyanin-producing yeast strains was carried out by EasyClone MarkerFree method [58]. The constructed yeast cells are listed in Additional file 1: Table S5.

For both betaxanthins and betacyanins production analysis, the starter cultures of constructed yeast strains were grown overnight in the mineral medium as described previously [63]. The next day, the cells were harvested and inoculated to freshly made MM (pABA^−^) medium, *i.e.*, modified mineral medium without p-aminobenzoic acid, to starting OD_600_ = 0.1. This medium consisted of 20 g/L glucose, 7.5 g/L (NH_4_)_2_SO_4_, 14.4 g/L KH_2_PO_4_, 0.5 g/L MgSO_4_·7H_2_O, 2 mL/L trace metal solution (3.0 g/L FeSO_4_·7H_2_O, 4.5 g/L ZnSO_4_·7H_2_O, 4.5 g/L CaCl_2_·2H_2_O, 0.84 g/L MnCl_2_·2H_2_O, 0.3 g/L CoCl_2_·6H_2_O, 0.3 g/L CuSO_4_·5H_2_O, 0.4 g/L Na_2_MoO_4_·2H_2_O, 1.0 g/L H_3_BO_3_, 0.1 g/L KI, and 19.0 g/L Na_2_EDTA·2H_2_O), and 1 mL/L vitamin solution (0.05 g/L D-biotin, 1.0 g/L D-pantothenic acid hemicalcium salt, 1.0 g/L thiamin–HCl, 1.0 g/L pyridoxin–HCl, 1.0 g/L nicotinic acid, and 25.0 g/L *myo*-inositol). Para-aminobenzoic acid was excluded from the medium to avoid interaction with betalain production.

### Constructing the yeast library

To construct the yeast library, the method described by Kildegaard et al. 2019 [42] was used. To do this, the parent strain ST8251 was grown in a 25 mL YPD medium supplemented with G418 for 4 h at 30 °C and 250 rpm, to get an optical density of 1.0–1.5. The cells were then harvested and transformed with the pooled DNA library using the standard lithium acetate method described in EasyClone MarkerFree method [58]. The pooled DNA library consisted of 10 μg of gRNA vector pCfB2310, ca. 5 pmol of P*tef1*-DOD-T*cyc1* and P*tdh3*-TyH-T*adh1*, and 15 pmol of the fragments for upstream- and downstream homology arms and Klura3 marker (Fig. 2). This mixture was purified by ethanol precipitation [64], and resuspended in 148 μL of water. The transformed cells were cultivated overnight in 25 mL of synthetic dropout medium without uracil (SC-ura) at 30 °C and 250 rpm. The culture was then diluted 1:10 into 5 mL of MM (pABA^−^) medium, with the rest of the culture mixed 1:1 with 50% glycerol and stored at – 80 °C. The next day, the culture was again diluted 1:50 into 5 mL MM (pABA^−^) medium and grown for 16 h. This culture was used for fluorescence-activated cell sorting (FACS).

### Flow cytometry and library sorting

To remove secreted betaxanthins, the cells were washed two times with PBS buffer (pH 7.5) by centrifuging at 3000 × *g* for 5 min. The cells were then resuspended in PBS buffer for analysis or sorting. The libraries of yeast cells were sorted using Sony SH800 Cell Sorter (Sony, Tokyo, Japan) to identify the most promising enzyme variants in regard to the desired phenotype. The measurements were performed using a 488 nm laser and a 525/50 band-pass filter. As cell doublets might significantly mislead the sorting experiments, events were first gated for live cells by linear alignment of FSC-height vs. FSC-area, and then gated for singlets by discriminating single and double cells in SSC-width vs. SSC-height. The gate sizes were set to capture approximately 40% and 80% of the population, respectively. Original library cells (named: Library 1, Fig. 2) passing the live and singlet selection gates were sorted for the top 7% of the fluorescence distribution. 10,000 events were sorted and collected to culture tube containing 2 mL SC-ura medium, and were grown overnight (named: Library 2, Fig. 2). The sorting process was repeated once more on these cultures, with selection gate for the top 4% of fluorescence distribution in live singlet cells to ensure for enrichment of high-fluorescent single cells. The second-round sorted cells (named: Library 3, Fig. 2) were plated on Nunc™ OmniTray™ single-well plates (Thermo scientific) containing MM (pABA^−^) agar medium, with a density of 3000–5000 events per plate. The plated cells were incubated at 30°C for 4–5 days, until single colonies were obtained. Based on visual selection, the most intense yellow colored single isolates were selected and proceeded with betaxanthins content analysis.

### Short-read (Illumina) genome resequencing

Strain ST8251 and the isolated singlets iso2, iso16 and iso21 were grown in 10 mL culture tubes containing 4 mL YPD medium supplemented with 20 g/L glucose as the carbon source. The cultures were incubated at 30 ˚C for two days and then the genomic DNA was isolated using the Quick-DNA™ fungal/Bacterial Miniprep kit (ZYMO Research, USA) according to the manufacturer’s instructions. The extracted DNA was quantified using a Qubit® 2.0 Fluorometer (Invitrogen, ThermoFisher Scientific). The isolated DNA was sequenced on MiSeq V2 (Illumina, San Diego, CA) with paired-end reads, 50-fold genomic coverage of the *S. cerevisiae* genome size (12 Mb). For all the strains, the reads were mapped using Bowtie2 [65] to the CEN.PK113-7D genome at NCBI (BioProject PRJNA393501) [66]. To allow mapping of the reads to the heterologous genes, an artificial chromosome consisting of each of the codon-optimized gene variants for DOD and TYH protein listed in Table 1 was added to the CEN.PK113-7D genome.

### Long-read (Nanopore) genome resequencing

For isolation of high quality ultra-high molecular weight DNA with long fragments, the genomic DNA isolation and purification method described by Denis et al. [67], followed by long-read sequencing using Nanopore GridION X5 (Oxford Nanopore Technologies, Oxford, UK) with a 50X coverage. For all four strains, the reads were mapped to each of the codon-optimized gene variants for DOD and TyH using Minimap2 v.2.25 [68, 69]. The reads mapped to each of the DOD and TyH variants were extracted using samtools v. 1.17 [70], and mapped back to the CEN.PK113-7D genome (NCBI, BioProject PRJNA393501) to identify the genomic regions of heterologous gene insertions.

### Disruption of transporter genes in betalain-producing strains

Plasmids for transporter gene disruption were constructed by amplifying the four desired disruption gRNAs for *QDR1*, *QDR2*, *ALP1* and *YJR015W* from a pooled transporter mutant library (Addgene accession numbers 153101–153106) previously described by Wang et al. [49] with primers adding 20 bp homology to the pTAJAK-71 backbone (Additional file 1: Table S4). The backbone was linearized by PCR and the two fragments were assembled using 2 × NEBuilder® HiFi DNA Assembly MasterMix (New England Biolabs). *E. coli* strain DH5α was transformed with the assembly mix and successful assembly was verified by Sanger sequencing. The *S. cerevisiae* strains were then transformed with the plasmid and selected on synthetic complete agar plates supplemented with 200 mg/L G418 (Sigma–Aldrich) for Cas9 maintenance and 100 mg/L nourseothricin (Sigma–Aldrich) for gRNA plasmid selection.

### Betaxanthins and betacyanins content analysis

For screening the DOD-TyH gene combinations, single isolates from Library 3 were cultivated overnight in 96-deep-well plate containing 400 µL MM (pABA^−^) with air-penetrable metal lid (EnzyScreen, The Netherlands) at 30 °C and 250 rpm. For betaxanthins content in other constructed yeast cells, the cells were cultivated in a similar way, but in 24-deep-well-plates containing 2 mL MM (pABA^−^). In both cases, the overnight grown cultures were diluted to fresh MM (pABA^−^) on next day, to get the starting OD_600_ = 0.1, and were incubated at 30 °C and 250 rpm for 48 h. The optical density (OD_600_) and fluorescence (485–515 nm) were measured in a plate reader (BioTek ELx 808) at 24 and 48 h, and the betaxanthins content is reported as fluorescence/OD_600_.

For betacyanins production, the cultivation was also done in 24-deep-well plates containing 2 mL MM (pABA^−^) with starting OD_600_ value of 0.1, terminated at 48 h. The cultures were then analyzed for cell growth (OD_600_), extracellular and total betanin content, and UV–vis spectra of the supernatant.

### HPLC analysis

Betanin and isobetanin quantification was done in Dionex Ultimate 3000 HPLC (Thermo Fisher Scientific), equipped with a Zorbax® column with particle size 5 μm. The column oven temperature was set at 30 °C and the flow rate to 1 mL/min, with 10 μL of sample injection. The absorbance at 390 nm, 410 nm, 480 nm and 540 nm was measured with a UV–Vis detector. Solvent A was 0.1% formic acid, and solvent B was acetonitrile. Solvent composition was initially A = 98.0%, and B = 2.0%, which was kept for 2 min. Then, solvent composition was changed following a linear gradient until A = 90.0%, and B = 10.0% at 5.0 min. In a second linear gradient, solvent composition changed until A = 85.0% and B = 15.0% at 8.0 min. At 8.2 min, solvent composition was increased to A = 2.0%, and B = 98.0%. The condition was kept constant until 9.5 min after which the initial composition was retrieved (A = 98.0%, B = 2.0%) and remained unchanged until the end of the run (11.5 min). Peak areas were used for compound quantification using external standard calibration method. Analysis of HPLC results was performed using the software Chromeleon 7 (ThermoFisher Scientific).

As pure betanin standard is not commercially available, we used the only commercial product available on the market, the red beet extract diluted with dextrin from Sigma–Aldrich [71] (product ID:901266-5G). By analyzing this sample, we observed an equimolar mixture of betanin and its C15-stereoisomer isobetanin, with retention times of 5.7 min and 6.1 min, respectively (Additional file 1: Fig. S7). To quantify the betacyanin content in this mixture, we used the Beer-Lambert equation by assuming the molar extinction coefficient *ε* = 6.5 × 10^4^ M^−1^ cm^−1^ for betanin [72]. The stock solution of red beet extract in MM (pABA^−^) medium was prepared in 10 g/L concentration, from which samples of 2 g/L, 1 g/L and 0.5 g/L were made. By reading the absorbance of these samples at λ = 536 nm in cuvettes with 1 cm path length, the betanin content in 1 g/L of red beet extract was calculated to be 0.837 mg/L (Table 3).

**Table 3:** Betanin content calculated for red beet standard solution (1:1 mixture of betanin and isobetanin) using the Beer-Lambert equation

Red beet samples	Absorbance_536nm_	Calculated betanin content (mg/L)
2 g/L	0.394 ± 0.001	3.337 ± 0.008
1 g/L	0.198 ± 0.000	1.674 ± 0.005
0.5 g/L	0.1 ± 0.001	0.847 ± 0.008

To determine the betanin content in supernatant of yeast cultures, 1 mL of cultivation broth was centrifuged at 10,000*g* for 10 min to precipitate all the cells and debris, and the supernatant was moved to amber-colored vials for HPLC analysis. For quantification of total betanin content (intracellular + extracellular), the cell cultures were extracted. To do this, 1 mL of cell culture was transferred into 2 mL microtube (Sarstedt) containing ca. 0.5 mL of 0.5–0.75 mm glass beads. The cells were then disrupted using the Precellys R 24 homogenizer (Bertin Corp.) in three cycles of 6500 RPM for 45 s, with cooling the tubes by placing on ice for at least 1 min in between each lysis cycle. After disruption, the tubes were centrifuged for 10 min at 10,000*g*. The supernatant was transferred to amber-colored vials for HPLC analysis. It worth mentioning that with this analysis method, the analytical sensitivity was as low as 0.5 mg/L of betanin/isobetanin.

### LC–MS analysis

The LC–MS/MS analysis was performed on a Vanquish Duo UHPLC binary system (Thermo Fisher Scientific, USA) coupled to UV detector (Thermo Fisher Scientific, USA) and to IDX-Orbitrap Mass Spectrometer (Thermo Fisher Scientific, USA). The analytes were separated using a Waters ACQUITY BEH C18 (10 cm × 2.1 mm, 1.7 μm) column equipped with an ACQUITY BEH C18 guard column kept at 40 C. The mobile phases consisted of MilliQ© water + O.1% formic acid (A) and acetonitrile + 0.1% formic acid (B). The initial composition was 2%B held for 0.8 min, followed by a linear gradient till 5% in 3.3 min, and to 100%B in 10 min held for 1 min before going back to initial conditions. Re-equilibration time was 2.7 min. The flow rate was set at 0.35 mL/min. The MS measurement was done in positive-heated electrospray ionization (HESI) mode with a voltage of 2500 V acquiring in full MS/MS spectra (Data dependent Acquisition-driven MS/MS) in the mass range of 70–1000 Da [73].

### Purification of UGT73A36

The gene coding for UGT73A36, codon-optimized for *S. cerevisiae*,was cloned by Gibson assembly into a pET-28a plasmid and fused to a N-terminal 6xHis-tag (pCfB11487). One Shot BL21 (DE3) competent cells (Thermo Fisher Scientific, USA) were transformed with the plasmid, resulting in strain ST12379. The strain was cultivated in 500 mL of 2xYT media at 37 °C, 200 rpm. Gene expression was induced at OD 0.5 with 250 mM IPTG and the cells were incubated for 16 h at 20 °C. After centrifugation, the cell pellet was resuspended in NaPO_4_ buffer (pH7) and stored at − 20 °C overnight. 20 mL DNAse (20 mg/mL) and TritonX100 (0.05%) was added to the thawed cells and the cells incubated for 20 min. The cells were lysed by sonification (4 cycles at 30 s: 70% amplitude, 30 s pulse, 30 s pause). After centrifugation (15000 × *g*, 4 °C, 30 min) to remove the cell debris, the supernatant was filtered with a 0.45 mm filter. Purification was performed with an ÄKTA Pure Protein Purification (Cytiva, USA) on a 1 mL HisTrap column (Cytiva, USA). After washing the column with buffer A (50 mM HEPES, 300 mM NaCl, pH 7.4), the sample was loaded on the column. After another washing step, elution of the protein of interest was performed running a gradient from 2 to 40% buffer B (50 mM HEPES, 300 mM NaCl, 500 mM imidazole, pH 7.4) in buffer A followed by 100% buffer B to remove all sample from the column. The fractions containing protein were pooled and up-concentrated to 1 mL using a 30 kDa centrifugal filter (Merck Millipore, USA) followed by 4 rounds of washing with buffer C (25 mM HEPES, 50 mM NaCl, pH 7.4) to remove imidazole from the purified protein. The resulting protein solution was analyzed on SDS-PAGE and the protein concentration determined using a BCA protein assay kit (Thermo Fisher Scientific, USA). The protein was aliquoted, frozen in liquid nitrogen and stored at − 80 °C.

### *In-vitro* glycosylation assay

Betanidin was obtained by deglycosylation of the betanin in beet root juice. 80 µL of β-glucosidase from almonds (Sigma–Aldrich) and 420 µL of 10 mM Na- ascorbate (pH 5.5) were added to 500 µL of sterile filtered beet root juice (ca. 200 mg/L betanin concentration) and incubated for 2 h at 37 °C. The Na-ascorbate ensures the stability of the formed betanidin. To verify the successful deglycosylation of betanin and formation on betanidin, the samples were quantified in HPLC, confirming that 94% of the (iso-)betanin has been deglycosylated to (iso-)betanidin. Betanidin concentration was determined by Beer-Lambert equation assuming a molar extinction coefficient *ε* = 5.4 × 10^4^ M^−1^ cm^−1^. The β-glucosidase was removed from the sample by size-exclusion, using a 30 kDa centrifugal filter unit (Merck Millipore, USA). The reaction mix for the glycosylation assay contained 140 mmol/L KPO_4_ buffer (pH 6), 3.2 mmol/L UDP-glucose (Sigma–Aldrich), ca. 20 µmol/L betanidin and ca. 50 µg (5 µL) purified UGT73A36 in a total volume of 50 µL. The reaction was started by addition of the enzyme and incubated at 30 °C for 16 h. For the control, H_2_O was added instead of purified UGT73A36. The reaction was stopped by keeping the mix at max. 4 °C before analysis by HPLC.

### Supplementary Information

**Additional file 1: Table S1**. Amino acid sequences and accession numbers for heterologous genes, together with corresponding codon-optimized nucleotide sequence of these genes used for integration to S. cerevisiae2. **Table S2**. List of the BioBricks used in this study11. **Table S3**. List of Primers used in this study.13 **Table S4**. List of plasmids used in this study15. **Table S5**. List of yeast strains constructed in this study16. **Figure S1**. The fluorescence of cell populations used for FACS, and also the cell color difference for ST10319 and Library3 on SC-ura plates18 **Figure S2**. Cultivation of multiple colonies obtained by integrating PgDODT732∆ together with two different TyH genes into genome of S. cerevisiae. The media is minimal media without pABA. The parent strain is CEN.PK113-7D. The strains were constructed using EasyClone toolbox, and the correct integration of fragments into genome was verified by colony PCR.19 **Figure S3**. Betalain production in yeast strains. The photos are taken after 48 hours of growth in MM (pABA-). The parent strain for integration of glucosyltransferases is iso2.20 **Figure S4**. MATGAT analysis for DOD enzyme variants21 **Figure S5**. MATGAT analysis for CYP76ADα (termed as TyH in this study) enzyme variants21. **Figure S6**. SDS-PAGE of fractions collected during purification of recombinantly expressed UGT73A36.22 **Figure S7**. HPLC-chromatogram and 3-D contour plot of betanin standard solution (1 g/L) from Sigma–Aldrich.23 **Figure S8**. Effect of TyH-DOD expression ratio on betaxanthins titer.24

## Data Availability

All data generated or analyzed during this study are included in this published article and its supplementary information files.

## Abbreviations

L-DOPA: L-3,4 dihydroxyphenylalanine
DOD: 4,5-Dopa-extradiol dioxygenase
TyH: Cytochrome P450 monooxygenase CYP76ADα
FACS: Fluorescence-activated cell sorting
UGT: UDP-glucosyltransferase
HPLC: High-performance liquid chromatography
MM (pABA^−^): Modified mineral medium without p-aminobenzoic acid
AA: Amino acid
IPTG: Isopropyl β-D-1-thiogalactopyranoside

## Footnotes

1 https://www.ncbi.nlm.nih.gov/assembly/GCF_000511025.2/.
