# Supplementary material for: Combinatorial engineering of betalain biosynthesis pathway in yeast Saccharomyces cerevisiae
Source: Biotechnol Biofuels Bioprod. 2023 Aug 17;16:128. doi: 10.1186/s13068-023-02374-4 (PMC10436450; doi:10.1186/s13068-023-02374-4)
Supplement: Supplementary file 1 — Additional file 1: Table S1. Amino acid sequences and accession numbers for heterologous genes, together with corresponding codon-optimized nucleotide sequence of these genes used for integration to S. cerevisiae2. Table S2. List of the BioBricks used in this study11. Table S3. List of Primers used in this study.13 Table S4. List of plasmids used in this study15. Table S5. List of yeast strains constructed in this study16. Figure S1. The fluorescence of cell populations used for FACS, and also the cell color difference for ST10319 and Library3 on SC-ura plates18 Figure S2. Cultivation of multiple colonies obtained by integrating PgDODT732∆ together with two different TyH genes into genome of S. cerevisiae. The media is minimal media without pABA. The parent strain is CEN.PK113-7D. The strains were constructed using EasyClone toolbox, and the correct integration of fragments into genome was verified by colony PCR.19 Figure S3. Betalain production in yeast strains. The photos are taken after 48 hours of growth in MM (pABA-). The parent strain for integration of glucosyltransferases is iso2.20 Figure S4. MATGAT analysis for DOD enzyme variants21 Figure S5. MATGAT analysis for CYP76ADα (termed as TyH in this study) enzyme variants21. Figure S6. SDS-PAGE of fractions collected during purification of recombinantly expressed UGT73A36.22 Figure S7. HPLC-chromatogram and 3-D contour plot of betanin standard solution (1 g/L) from Sigma–Aldrich.23 Figure S8. Effect of TyH-DOD expression ratio on betaxanthins titer.24 [file 13068_2023_2374_MOESM1_ESM.docx]

Additional file 1

Combinatorial engineering of betalain biosynthesis pathway in yeast *Saccharomyces cerevisiae*

Mahsa Babaei ^a^, Philip Tinggaard Thomsen ^a^, Jane Dannow Dyekjær ^a^, Christiane Ursula Glitz ^a^, Marc Cernuda Pastor ^a^, Peter Gockel ^a^, Johann Dietmar Körner ^a^, Daniela Rago ^a^, Irina Borodina ^a^*

^a^ The Novo Nordisk Foundation Center for Biosustainability, Technical University of Denmark, Kemitorvet Building 220, DK-2800 Kgs. Lyngby, Denmark

*Corresponding author: Irina Borodina, orcid.org/0000-0002-8452-1393

Email: [irbo@biosustain.dtu.dk](mailto:irbo@biosustain.dtu.dk)

[Table S1: Amino acid sequences and accession numbers for heterologous genes, together with corresponding codon-optimized nucleotide sequence of these genes used for integration to *S. cerevisiae* 2](#_Toc75168182)

[Table S2: List of the BioBricks used in this study 11](#_Toc75168183)

[Table S3: List of Primers used in this study. 13](#_Toc75168184)

[Table S4: List of plasmids used in this study 15](#_Toc75168185)

[Table S5: List of yeast strains constructed in this study 16](#_Toc75168186)

[Figure S1: The fluorescence of cell populations used for FACS, and also the cell color difference for ST10319 and Library3 on SC-ura plates 18](#_Toc137209874)

[Figure S2: Cultivation of multiple colonies obtained by integrating *Pg*DOD^T732∆^ together with two different TyH genes into genome of *S. cerevisiae*. The media is minimal media without pABA. The parent strain is CEN.PK113-7D. The strains were constructed using EasyClone toolbox, and the correct integration of fragments into genome was verified by colony PCR. 19](#_Toc137209875)

[Figure S3: Betalain production in yeast strains. The photos are taken after 48 hours of growth in MM (pABA^-^). The parent strain for integration of glucosyltransferases is iso2. 20](#_Toc137209876)

[Figure S4: MATGAT analysis for DOD enzyme variants 21](#_Toc137209877)

[Figure S5: MATGAT analysis for CYP76ADα (termed as TYH in this study) enzyme variants 21](#_Toc137209878)

[Figure S6: SDS-PAGE of fractions collected during purification of recombinantly expressed UGT73A36. 22](#_Toc137209879)

[Figure S7: HPLC-chromatogram and 3-D contour plot of betanin standard solution (1 g/L) from Sigma-Aldrich. 23](#_Toc137209880)

[Figure S8: Effect of TYH-DOD expression ratio on betaxanthins titer. 24](#_Toc137209881)

Table S1: Amino acid sequences and accession numbers for heterologous genes, together with corresponding codon-optimized nucleotide sequence of these genes used for integration to *S. cerevisiae*

| **Gene** | **Sequence** |
| --- | --- |
| *MjDOD*   (B6F0W8.1) | MKGTYYINHGDPLMYLKKHIKLRQFLEGWQENVVIEKPKSILIISAHWDTNVPTVNFVEHCDTIHDFDDYPDPLYQIQYRAPGAPNLAKKVEELLKESGMECEIDTKRGLDHAAWFPLMFMYPEANIPICELSVQPSKDGIHHYNVGKALSPLLQQGVLIIGSGGTVHPSDDTPHCPNGVAPWAIEFDNWLEDALLSGRYEDVNNFKKLAPNWEISHPGQEHLYPLHVALGAAGKNPKTQLIHRSWAANGVFGYSTYNFTPTTQKTD |
|  | atgaagggaacctactacatcaaccacggtgacccattgatgtacttgaagaagcacatcaagttgagacaattcttggaaggttggcaagaaaacgttgttatcgaaaagccaaagtctatcttgatcatcagtgcacactgggacaccaacgttccaaccgttaacttcgttgaacactgtgacaccatccacgacttcgacgactacccagacccattgtaccaaatccaataccgcgctccaggtgctccaaacttggctaagaaggttgaagaattgcttaaggaatctggtatggaatgtgaaatcgacaccaagagaggtttggaccacgctgcttggttcccattgatgttcatgtacccagaagctaacatcccaatctgtgaattgtctgttcaaccatctaaggacggtatccaccactacaacgttggtaaagctttgtctccattgttgcaacaaggtgttttgatcatcggttctggtggaaccgttcacccatctgacgacaccccacactgtccaaacggtgttgctccatgggctatcgagttcgacaactggttggaagacgctttgctctcgggtagatacgaagacgttaacaacttcaagaagttggctccaaactgggaaatctctcacccaggtcaagaacacttgtacccattgcacgttgctttgggtgctgctggtaagaacccaaagacccaattgatccaccggtcttgggctgctaacggtgttttcggttactctacctacaacttcaccccaaccacccaaaagaccgacgtaa |
| *BvDOD1*  (Q70FG7.1) | MGSEDNIKETFFISHGTPMMAIDDSKPSKKFLESWREKIFSKKPKAILVISAHWETDQPSVNVVDINDTIYDFRGFPARLYQFKYSAPGSPELANRIQDLLAGSGFKSVNTDKKRGLDHGAWVPLMLMYPEADIPVCQLSVQSHLDGTHHYKLGQALAPLKDEGVLIIGSGSATHPSNGTPPCSDGVAPWAAAFDSWLETALTNGSYEEVNKYETKAPNWKLAHPWPEHFYPLHVAMGAAGENSKAELIHNSWDGGIMSYGSYKFTST |
|  | atgggttctgaagataatattaaagaaacatttttcatctctcatggcactccaatgatggctattgatgattccaagccatctaagaagtttttggaatcttggagagaaaagattttctctaagaaaccaaaagctatcttggttatctctgcccattgggaaactgatcagccgtccgttaatgttgttgatattaatgatactatttacgatttcaggggttttccagctagattgtaccaatttaagtactcagctccaggttctcccgaattagctaacagaattcaagatttgttagccggctctggttttaaatctgttaatactgataagaaaaggggtctcgatcatggtgcttgggttccattgatgttaatgtatccagaagctgatattccagtttgtcaattgtccgttcaatcacatttggatggtactcatcattacaaattgggtcaggctttagctccattgaaagatgaaggcgtccttattattggttctggttctgctacccatccatccaatggtactccaccatgttctgatggtgttgccccatgggctgctgcttttgattcttggttagagactgccttgactaatggttcttacgaagaagttaacaagtatgagactaaagctccaaactggaagttggcccatccatggccagaacatttttacccattacatgtcgccatgggtgctgctggcgaaaattctaaagctgaattgattcataattcttgggacggtggtattatgtcttatggttcttataaatttacttctacttaa |
| *BgDOD1*  (ASW22755.1) | MGGEKKMKGTYYLAHGDPIMYINKSIKLRHFLEGWKENVLTEKPKCILVISAHWDTDVPTVNLVEQCDTIHDFDDYPDPLYQIKYPAPGAPKLAMKVQELLKGGGFKCEVDTKRGLDHAVWFPLMFMYPEADIPICELSIQTSKDGTHHYNVGKALSPLLNDDVLIIASGGAVHPSDDTPHFPNGVAPWALEFDNWLEGALLSGRYEDVKEFKKLAPNWEISHPGQEHLYPLHVALGAAGNNVKTELIHQTWAANGVFGYSSYKFTST |
|  | atgggtggtgaaaagaaaatgaaaggtacttattatttggcccacggtgatccaattatgtatattaataaatctattaaattgagacacttcttggagggttggaaagaaaatgtcttgactgaaaagccaaaatgtattttggtcatttctgcacactgggatactgatgttccaaccgttaacttagttgaacaatgtgatacgattcatgattttgatgattatccagatccattgtatcagattaaatatccagctccaggtgctccaaagttggctatgaaagttcaagaattgttaaaaggtggcggctttaagtgtgaagttgatactaaaagaggcttggatcacgctgtttggtttccattaatgtttatgtacccagaagccgatattccaatttgtgaattgtccattcaaacttctaaagatggtactcatcactacaacgtcggtaaggctttgtctccattgttgaatgatgacgtcttgattattgcttctggtggcgctgttcatccatctgatgatactccacattttccaaatggtgttgccccctgggctttggaatttgataattggttggaaggtgctttgttgtctggtagatatgaagatgttaaggagtttaagaaattggctccaaattgggaaatcagccatccaggtcaagaacatttgtatccattacacgtcgctctgggtgctgctggtaataatgttaagactgaattgatccatcaaacttgggctgctaatggtgtttttggttattcttcttataaatttacttctacttaa |
| *PgDOD*  (CAE45178.1) | MGVGKEVSFKESFFLSHGNPAMLADESFIARNFLLGWKKNVFPVKPKSILVVSAHWETDVPCVSAGQYPNVIYDFTEVPASMFQMKYPAPGCPKLAKRVQELLIAGGFKSAKLDEERGFDHSSWVPLSMMCPEADIPVCQLSVQPGLDATHHFNVGRALAPLKGEGVLFIGSGGAVHPSDDTPHWFDGVAPWAAEFDQWLEDALLEGRYEDVNNYQTKAPEGWKLAHPIPEHFLPLHVAMGAGGEKSKAELIYRTWDHGTLGYASYKFTSI |
|  | atgggtgtaggtaaagaagtttcttttaaagaatccttcttcttgtctcacggtaatccagctatgttggccgacgaatcctttattgctagaaatttcttactgggctggaaaaagaatgtttttccagttaaacccaaatctattctggttgtttctgctcactgggaaactgatgttccatgtgtttctgctggtcaataccccaacgtgatttatgattttactgaagttccagcttccatgttccaaatgaaatatccagctccaggttgtccaaaattggctaaaagagttcaagaattgttgattgcaggtggctttaaatctgctaagctggatgaagaaaggggttttgatcattcttcttgggttcccttatctatgatgtgtccagaagctgatattccagtttgtcagttatctgttcaaccaggtttggatgctactcatcattttaatgttggtagagctttagccccattgaaaggtgaaggtgttttatttattggctccggtggcgctgttcatccatctgatgatactccacattggtttgatggcgttgctccatgggctgctgaattcgaccaatggttggaagatgctttgttagaaggtagatatgaagacgtcaataattatcaaactaaagctccagaaggttggaaattggctcatccaattccagagcacttcttgccattgcatgttgctatgggtgctggcggcgagaaatctaaagctgaattgatttatagaacttgggatcatggtaccttaggttatgcttcttataaatttacttctatttaa |
| *PgDOD**  (mutated version: T_732_Δ) | MGVGKEVSFKESFFLSHGNPAMLADESFIARNFLLGWKKNVFPVKPKSILVVSAHWETDVPCVSAGQYPNVIYDFTEVPASMFQMKYPAPGCPKLAKRVQELLIAGGFKSAKLDEERGFDHSSWVPLSMMCPEADIPVCQLSVQPGLDATHHFNVGRALAPLKGEGVLFIGSGGAVHPSDDTPHWFDGVAPWAAEFDQWLEDALLEGRYEDVNNYQTKAPEGWKLAHPIPEHFLPLHVAMGLAARNLKLN*~~FIELGIMVP*VMLLINLLLF~~ |
|  | atgggtgtaggtaaagaagtttcttttaaagaatccttcttcttgtctcacggtaatccagctatgttggccgacgaatcctttattgctagaaatttcttactgggctggaaaaagaatgtttttccagttaaacccaaatctattctggttgtttctgctcactgggaaactgatgttccatgtgtttctgctggtcaataccccaacgtgatttatgattttactgaagttccagcttccatgttccaaatgaaatatccagctccaggttgtccaaaattggctaaaagagttcaagaattgttgattgcaggtggctttaaatctgctaagctggatgaagaaaggggttttgatcattcttcttgggttcccttatctatgatgtgtccagaagctgatattccagtttgtcagttatctgttcaaccaggtttggatgctactcatcattttaatgttggtagagctttagccccattgaaaggtgaaggtgttttatttattggctccggtggcgctgttcatccatctgatgatactccacattggtttgatggcgttgctccatgggctgctgaattcgaccaatggttggaagatgctttgttagaaggtagatatgaagacgtcaataattatcaaactaaagctccagaaggttggaaattggctcatccaattccagagcacttcttgccattgcatgttgctatggggctggcggcgagaaatctaaagctgaattgatttatagaacttgggatcatggtaccttaggttatgcttcttataaatttacttctatttaa |
| *SoDOD*  (XP_021836119.1) | MILHNFQQPSTLLLQIPSFSATPEIKRTFLKSKFPKNSNLKIMAGQESIKETFFISHGTPMMAIDESKPSRKFLESWREKIYSKKPKAILVISAHWETDSPSVNSVDVNDTVYDFGGFPARLYQFKYPAPGFPDLAKRVQELLTASGFQSVHTDKKRGLDHGAWVPLMLMYPEADIPVCQLSVQSHLDGKYHFNLGRALAPLKDEGVLIIGSGSATHPSNGTPHCNDGVAPWAADFDLWLETALTSGRYEEVNKCERKAPNWKLAHPWPEHFYPLHVAMGAAGENSKAELIHNSWDHGTMSYGSYKFSPN |
|  | atgattttgcataattttcaacaaccatctactttgttgctgcaaattccatctttttctgctactccagaaatcaaaaggactttcttgaagtccaagttccctaaaaattctaatttgaaaattatggccggtcaagagtccattaaagaaacatttttcatttctcatggtactccaatgatggctattgatgaatctaaaccttcccgcaaatttttagaatcttggagagaaaagatttattcaaagaagccaaaagctattttggttatctctgcccattgggaaactgattctccctctgttaattctgttgatgttaatgatactgtttatgactttggcggctttccagctagattgtatcaattcaagtaccccgccccaggttttccagatttagctaagagagttcaagagttattgactgcttctggttttcagtctgttcacactgataagaaaaggggtttggaccacggtgcctgggttccattgatgttgatgtatccagaagctgacatcccagtttgtcaattgtctgttcaatctcacttggatggtaaatatcattttaatttaggtagggccttagccccattaaaagatgaaggtgttttgattattggctctggttcagctactcatccatctaacggtactccacactgtaacgatggtgttgctccatgggctgccgatttcgacttgtggctagaaactgctttgacttctggccgttacgaagaagttaataaatgtgaaagaaaggctccaaattggaagcttgctcatccatggccagaacatttctatccattgcatgttgctatgggtgctgccggtgagaattccaaagctgaattaattcataattcttgggatcacggtactatgtcttatggttcttataaattttctccaaattaa |
| *AtDOD*  (AJW81119.1) | MGSQEIIKETFFISHGTPRMTIEASKPARKFLESWRDKIYFKKPKAILVISAHWETDFPSVNAVDINDTIYDFYGFPAPMYQFKYPAPGSPDLAQRVQELLTASGFKSVNVDKKRGLDHGAWVPLMLMYPNADIPVCQLSVQSHLDGMYHYKLGRALAPLKEEGVLIIGSGSATHPSNNTPHYYDGVAPWAADFDHWLETALTNGSYEEVNKCESKAPNWKLAHPWPEHFYPLHVAMGAAGENWKAELIHNSWDHGTMSYGSYKFVSS |
|  | atgggttctcaagaaattattaaagaaactttctttatttctcacggcactccaagaatgactattgaagcttctaaacccgctagaaaatttctagaatcttggagagataagatctactttaagaaaccaaaagctatcctggttatctctgcccattgggaaactgatttcccatctgttaatgcagtggatattaatgatactatctacgatttctatggctttccagctccaatgtaccaattcaaatatcctgctccaggttctccagatttggcccagagagttcaagaattgttgactgcttctggttttaagtccgtcaatgttgataagaaaagaggtttagatcatggcgcttgggttccattgatgttaatgtatccaaacgctgatattccagtttgtcaattatctgttcaatctcacttggatggtatgtatcattacaaattgggtagagctttagctcccctgaaagaagaaggtgttttaattattggttctggttctgccactcacccatctaataatactccacattattacgacggcgttgctccatgggctgctgattttgatcattggttggaaaccgctttgacaaatggttcttacgaagaggttaataaatgtgaatctaaagctccaaattggaagttggctcatccatggccagaacatttctacccactacatgttgctatgggtgctgctggtgagaattggaaagctgaattgattcataattcttgggatcatgggactatgtcttatggttcttataaatttgtttcttcttaa |
| *AhDOD*  (ADZ48644.1) | MGSQEIIKETFFISHGTPRMTIEESKPARKFLESWRDKIYCKKPKAILVISAHWETDFPSVNAVDINDTIYDFYGFPAPMYQFKYPAPGSPDLAQRVQELLTASGFKSVNVDKKRGLDHGAWVPLMLMYPNADIPVCQLSVQSHLDGMYHYKLGRALAPLKEEGVLIIGSGSATHPSNSTPHYYDGVAPWAADFDQWLETALTNGSYEEVNKCERKAPNWKLAHPWPEHFYPLHVAMGAAGENWKAELIHNSWDHGTMSYGSYKFVSS |
|  | atgggttctcaagaaattattaaagaaactttctttatttctcacggcactccaagaatgactattgaagaatctaagccagctagaaagttcttggaatcttggagagataagatttattgtaagaaaccaaaagctattttggtcatttccgctcattgggaaactgatttcccatccgttaatgctgttgatattaatgatactatttacgatttctatggttttccagcaccaatgtatcaattcaaatatccagctccaggttctcccgatttggctcaaagggttcaagaattgttgactgcttctggtttcaaatctgttaatgttgataaaaagagaggtttggatcatggcgcttgggttccattgatgttgatgtacccaaatgctgatattccagtttgtcaattatctgttcaatcccatttggatggtatgtatcattacaagttgggtagagctttagctccattgaaagaggaaggcgtcttgattattggttctggttccgctactcatccctctaattctactccacattattatgatggcgttgctccatgggctgctgattttgatcaatggttggaaactgctttaactaatggttcttatgaagaagtcaataaatgcgagagaaaagctccaaattggaaattggctcatccatggccagaacatttttaccccttacatgttgctatgggtgctgctggtgagaattggaaagctgaattgattcataattcatgggatcatggtactatgtcttatggttcttataaatttgtttcttcataa |
| *PaDOD*  (BAH66635.1) | MDVKDMIRETFYISHGTPMMAINKSVPARSFLKGWREEVYSKKPKSILVISAHWETDLPTISAVNHSDLIYDFYGFPAPMYQLKYPAPGAPDLATRVQELLTVSGFKCALDKKRGLDHGSWVPLMFMYPDANIPVCQLSLQSHLDGTHHYKLGRALAPLKEEGVLVIGSGSSVHPSNDTPHAVGVAPWAAEFDNWLEEALTSGRYEDVNNYQTKAPNWKIAHPWPEHFYPLHVAMGAAGENAKAELIHRSWEHGTLGYACYKFTSS |
|  | atggatgttaaagacatgattagagaaactttttatatttcccacggtactccaatgatggctattaataagtctgttccagctagatcatttttgaaaggttggagagaagaagtctattctaaaaagccaaaatctatcttggtgatttccgctcattgggaaactgatttaccaacaatttctgcagtgaatcattctgacttgatttacgatttttatggttttccagccccaatgtatcagttgaaatatccagctccaggtgctccagacttggctaccagagttcaagaattattaactgtttctggtttcaagtgcgctttggataagaaaagaggtttggatcacggttcttgggttccattgatgtttatgtatccagatgccaatattccagtttgtcaattgtctctgcagtctcacttggatggtactcatcattataagttaggtagggctttggcaccattgaaagaagaaggtgttttggtcattgggtctggttcttctgttcatccatctaatgacaccccacatgctgttggtgttgctccatgggctgccgagttcgataattggttggaagaagctttaacctctggcagatacgaagatgttaataattatcaaaccaaagctccaaactggaaaattgctcatccatggcccgaacacttttatccattgcatgttgctatgggtgctgccggtgaaaatgccaaagctgaattgattcatagatcttgggagcatggtaccttgggttatgcttgttataaatttacttcttcataa |
| *SsDOD*  (ACO59903.1) | MGSNNNNEKIKETFFLSHGTPMMIIEESQPARKFLESWTEKIYSKKPKAILVISAHWETDHPAVTSVEVNDTIHDFYGFPAPMYQFKYPAPGSPDLAKRVQELLTASGFKSVQTDKKRGLDHGAWVPLMFMYPEPEIPVCQLSVQSHLDGKFHYNLGRALAPLKDEGVLIIGSGSATHPSNATPHSYDGVAPWAADFDCWLETSLTNGRYEEVNKCETKAPNWKLAHPWPEHFYPLHVAMGAAGEDWKAELIHNSWDHGTMSYGSYKFTSS |
|  | atgggttctaataataataatgaaaagattaaagagactttctttttgtctcatggtactccaatgatgattatcgaggaatctcaaccagctagaaaatttttggagtcttggaccgagaaaatttattccaagaaaccaaaagctattttggttatctctgcccattgggaaactgatcatccagctgtcacctctgttgaggttaatgatactattcatgatttttatgggtttcccgctccaatgtatcaatttaaatatccagctcccggttccccagatttggctaaaagagttcaagaattattgaccgcatccggttttaaatctgttcaaactgataagaagcgcggcttagatcatggtgcttgggttcccttgatgtttatgtacccagaaccagaaattccagtctgtcaattgtccgttcaatctcatttagatggcaagtttcattataatttaggtagagccttagctcccttgaaggatgaaggtgttttgattattggctctggctctgctactcatccatctaatgctactccccattcctatgacggtgttgctccatgggctgctgacttcgattgttggttagaaacttctttgactaacggtagatatgaagaggttaataaatgtgaaaccaaagctccaaattggaaattggctcatccatggccagaacacttttatccattgcatgttgctatgggggccgctggcgaagattggaaagctgaattgattcataattcctgggatcacggtactatgtcttacggttcctataaattcacttcttcataa |
| *BgDOD2*  (BAG80687.1) | MGGEKKMKGTYYIAHGDPIMYINKSIKLRHFLEEWKENVVMEKPICILVISAHWDTDVPTVNLVEHCDTIHDFDDYPDPLYQIKYPAPGAPKLAMKVQELLKGGGFKCEVDTKRGLDHAAWFPLMLMYPEADIPICELSVQTNKDGTHHYNLGKALSPLLNDDVLIIGSGGAVHPSDDTPHCPNGVAPWALQFDNWLEDALLSGRYEDVKEFKKMAPNWEISHPGQEHLYPLHVALGAAGNNVKTELIHQTWAANGVFGYSSYKFTST |
|  | atgggtggtgaaaagaaaatgaaaggtacttattatattgctcatggtgacccaattatgtatattaataagtctattaaattgaggcattttctagaagaatggaaagaaaatgttgtcatggaaaaaccaatttgtattttagttatttctgctcattgggatactgatgtcccaacagtcaatttggttgaacattgcgacactattcacgactttgatgattatcccgatccattgtatcaaattaaatatccagctccaggtgctcccaaattggccatgaaagttcaagaattgttgaagggtggtggtttcaaatgtgaagttgatactaaaagaggcttagaccatgctgcttggtttccattaatgctgatgtatccagaagctgatattccaatttgtgaattgtctgttcaaaccaacaaagatggtactcatcattataacttgggtaaagctttgtctccattactgaatgatgacgttctgattattggttctggtggtgccgttcatccttctgatgatactccacattgtccaaatggtgtcgctccctgggctttgcaatttgataattggttggaagacgccttgctatctggtaggtatgaagacgttaaagaatttaaaaagatggcccctaattgggaaatctctcatccaggtcaagaacatctctatcccttgcacgttgctttgggtgctgctggtaacaacgttaaaactgaattaattcatcaaacatgggctgctaacggtgtttttggttattcttcttataaatttacttctacttaa |
| *BvDOD2*  (AET43293.1) | MKMMNGEDANDQMIKESFFITHGNPILTVEDTHPLRPFFETWREKIFSKKPKAILIISGHWETVKPTVNAVHINDTIHDFDDYPAAMYQFKYPAPGEPELARKVEEILKKSGFETAETDQKRGLDHGAWVPLMLMYPEADIPVCQLSVQPHLDGTYHYNLGRALAPLKNDGVLIIGSGSATHPLDETPHYFDGVAPWAAAFDSWLRKALINGRFEEVNIYESKAPNWKLAHPFPEHFYPLHVVLGAAGEKWKAELIHSSWDHGTLCHGSYKFTSA |
|  | atgaaaatgatgaatggtgaagatgctaatgatcaaatgattaaagaatctttcttcattactcatggtaacccaattttaactgttgaagatactcatccattgagaccattctttgaaacttggagagaaaagatttttagtaagaagccaaaagctattttgattatttccggccactgggaaactgttaaaccaactgttaatgccgtccatatcaatgatactattcatgattttgatgattatccagctgccatgtatcaatttaaatatccagctccaggcgagccagaattagctagaaaagttgaagaaattttgaaaaagtccggtttcgaaactgctgaaactgatcaaaagagaggtttggatcatggtgcctgggttccattgatgttgatgtatccagaagctgatatcccagtctgtcaattatctgttcaaccacatttagatggtacttaccattataatttgggtagagccctggctcccttgaaaaatgatggtgtattgattattggttccggttctgccactcacccattggatgaaaccccacattattttgatggtgttgctccatgggctgctgcttttgattcttggttgagaaaagccttgattaacggtagattcgaagaagttaatatttatgaatctaaagctcccaattggaaattggctcatccattcccagaacacttttatccattgcatgttgttttaggtgctgctggtgaaaaatggaaagctgaattaatccattcctcctgggatcatggtactttgtgtcatggttcttataaatttacttctgcttaa |
| *BvDOD3*  (AET43287.1) | MKMMNGEDATDQMIKESFFITHGNPILTVEDTHPLRPFFETWREKIFSKKPKAILIISGHWETVKPTVNAVHINDTIHDFDDYPAAMYLFKYPAPGAPELARKVEEILKKSGFETAETDEKRGLDHGAWVPLMLMYPEADIPVCQLSVQPHLDGTYHYNLGRALAPLKNDGVLIIGSGSATHPLDETPHYFDGVAPWAAAFDSWLRKALINGRFEEVNIYETKAPNWKLAHPFPEHFYPLHVVLGAAGEKWKAELIHSSWDHGTLCHGSYKFTSA |
|  | atgaaaatgatgaatggtgaagatgctactgaccaaatgattaaggaatcatttttcattactcacggcaacccaattttgactgttgaagatactcatcccttgagacccttctttgaaacttggagagagaaaatttttagtaaaaagccaaaagctattttgatcatttccggtcattgggaaactgttaagccaaccgttaatgccgttcatattaatgataccattcatgattttgatgattatccagctgctatgtatttgtttaaatacccagctccaggcgccccagaattagctagaaaagttgaagaaattttgaaaaagtctggttttgaaactgctgaaactgatgagaagagaggtttggatcatggtgcttgggttccattgatgttgatgtatccagaagctgatattcccgtttgtcagttatctgttcaaccacacctggatggtacttatcattataatttgggtagagctttggccccgttgaagaatgatggtgttttgattattggttccggtagcgctactcatccattagatgaaactccacattattttgatggcgttgctccatgggctgctgctttcgattcttggctgagaaaagctttgattaatggtaggtttgaagaagttaacatttatgagactaaagcccccaattggaaattggctcatccattcccagaacatttctacccattgcatgttgttttaggtgctgctggtgaaaaatggaaagctgaattaattcattcttcttgggatcacggtactttgtgtcatggctcttataaattcacctctgcttaa |
| *BvCYP76AD^W13L^*  (AET43289.1) | MDHATLAMILAILFISFHFIKLLFSQQTTKLLPPGPKPLPIIGNILEVGKKPHRSFANLAKIHGPLISLRLGSVTTIVVSSADVAKEMFLKKDHPLSNRTIPNSVTAGDHHKLTMSWLPVSPKWRNFRKITAVHLLSPQRLDACQTFRHAKVQQLYEYVQECAQKGQAVDIGKAAFTTSLNLLSKLFFSVELAHHKSHTSQEFKELIWNIMEDIGKPNYADYFPILGCVDPSGIRRRLACSFDKLIAVFQGIICERLAPDSSTTTTTTTDDVLDVLLQLFKQNELTMGEINHLLVDIFDAGTDTTSSTFEWVMTELIRNPEMMEKAQEEIKQVLGKDKQIQESDIINLPYLQAIIKETLRLHPPTVFLLPRKADTDVELYGYIVPKDAQILVNLWAIGRDPNAWQNADIFSPERFIGCEIDVKGRDFGLLPFGAGRRICPGMNLAIRMLTLMLATLLQFFNWKLEGDISPKDLDMDEKFGIALQKTKPLKLIPIPRY |
|  | atggatcacgcaacattagctatgatattagcaattttgtttatttcatttcattttattaaattattattctcacaacaaacaacaaaattgttacctccaggtcctaaacctttaccaattattggtaatatcttggaagtaggaaagaagcctcatagatcatttgcaaatttggctaagatccacggtcctttaatctctttgaggttaggatcagttaccaccattgttgtatcatctgctgatgtcgccaaagagatgttcttgaagaaggatcaccctttatcaaacaggacaattccaaattcagtaactgctggtgaccaccacaaattgactatgtcttggttgcctgtttcacctaaatggagaaacttcagaaagataactgctgttcatttattgtctccacaaagattagatgcatgccagacctttagacatgcaaaggttcagcaattatatgaatacgttcaagagtgcgctcagaagggtcaggcagttgacattggtaaagctgccttcacaacttctttgaatttattgtctaaattattcttttctgttgaattggcccatcataaatcacatacctctcaagagtttaaggaattaatttggaatattatggaagacatcggtaaaccaaactacgccgattacttcccaattttaggttgcgtcgatccatcaggaattagaagaaggttggcatgttcttttgataagttaattgcagtatttcaaggaataatatgtgagaggttagctccagattcatcaactacaactactacaactacagatgatgtcttggacgtattattgcaattgtttaagcagaatgaattaactatgggagagattaaccacttgttagtcgatattttcgatgccggtactgatactacatcatctacctttgagtgggttatgactgagttaataagaaaccctgaaatgatggaaaaggcccaagaggaaataaaacaggtattgggtaaggataagcaaatccaagagtcagacataataaatttgccatacttgcaagcaatcattaaggaaactttgaggttgcacccacctacagttttcttgttgcctagaaaagccgacacagacgtagaattgtacggatacattgttccaaaggatgctcaaattttggtaaacttgtgggccataggtagagatccaaatgcttggcaaaatgccgatattttctcacctgaaagatttataggttgtgagattgatgtaaagggaagagattttggtttattgccttttggtgctggtagaagaatttgccctggtatgaatttggccataaggatgttgacattgatgttggccacattattgcaattcttcaactggaaattagagggagacatttcacctaaagatttggacatggatgaaaagtttggtatagccttgcagaaaactaaacctttaaaattgatcccaattccaagatactaa |
| *BaTyH*  (AJD87470.1) | MDNTTLAILLSTSYFIIYLIITKLGFHYKVLPKNPKQRLRLPPGPKPLPIIGNVLELGSKPHRSFTNLAKVHGPLISLRLGSVTTIIVSSSHVAKEMFLKNDQSLSSNRTIPHSVTAGDHHKLTMSWLPVSPKWRSFRKITTFHLLSPQRLDACCSLRQAKVQQLFEYVLQCSRTGQPVDIGKAAFTTSLNLLSKLFFSLELAHHRSTKSQEFKDLIWNIMEDIGKPNIADHFPCLKYFDPSGIRRRLASSFERLIEVFQDIIRQRMSLSFGSSHNNDVLDVLLGLYNQKELTMDEINHLLVDIFDAGTDTTSSTFEWSMAELMKNRRIMEKAQAEILHVLGKNSYIQESDISKLPYLRAIIKETLRLHPPTVFLLPRKADADVELYGYVVPKDAQILVNLWALGRDPAVWENPDEFSPDRFMGSEIDVKGRDFGLLPFGAGRRICPGMNLAIRMLTLMLATLLRSFDWKLPEGEAPAQLDMDEKFGIALQKTTPLKIIPIFKNSI |
|  | atggataatactactttggctatcttactatccactagctattttattatctacttgatcattactaaattagggttccattataaggtcttgccaaagaatccaaagcagaggcttagattaccaccaggtccaaagccactgccaatcataggtaatgttttggaattaggttctaagccccacagaagttttactaatttagctaaagttcatggacccttgatttctttaagattgggctctgtcactactattattgtttcatcttctcatgttgctaaagaaatgtttttgaaaaatgatcagtcattgtctagcaatagaactattccacattctgtcaccgctggtgatcatcataaattgacaatgtcttggttaccggtttctcccaaatggagatcattcagaaaaattactacttttcatttgctatctccacagaggttggatgcttgttgttctttgcgccaagctaaagtccaacaattgtttgaatacgttttacagtgtagcagaactggtcaaccagttgatattggaaaagcagcctttactacctctttaaatttgttgtctaagctcttcttttctttggaattagctcatcatcggtctacaaaatctcaagaatttaaagatttaatttggaatattatggaggatattggcaagccaaacattgctgaccattttccctgtttaaaatattttgatccatctggtattaggcgcaggttagcttcttcatttgaaagattgattgaggttttccaagatataattagacaacggatgagcttgtcttttggttcttctcacaataatgatgtcttggatgttttgttaggtttatataaccagaaggaacttacaatggacgaaattaaccatctcttggttgatatttttgacgctggtactgataccacttctagtacttttgaatggtcaatggctgaattaatgaaaaatagaagaattatggaaaaggctcaagcagaaattttacacgttttgggcaaaaattcatacatccaagaatctgatatttctaaattgccctatttgagagctattattaaagaaactttaaggctgcatccacctactgtctttttgctaccaagaaaagctgacgctgatgttgaattgtatggttacgttgtgccaaaggatgctcaaattttggttaacctatgggccttgggtagagatccagctgtttgggaaaatccagatgaattttctccagataggtttatgggttctgagattgatgtcaaaggtagagattttggtctattgccatttggtgctggtagaagaatctgccccggtatgaatttagctattagaatgctaacattaatgttagccacattgttaagatcctttgattggaagttgcctgaaggtgaagctccagcacagctggatatggatgaaaaatttggaattgcattacagaagactactccattgaaaattattccaatttttaaaaattcaatttaa |
| *CbTyH*  (AJD87468.1) | MDYTTLVMILSIVFFCYNLFNLLFTRKNTKLPPGPKTIPIFGNIFELGKKPHQSFANLAKIHGPLMSLKLGSVTTIVVSSAEVAREMFLKNDQLLSNRTVPNSVTAGDHHKTTMSWLPVSQKWRNFRKITAVHLLSPQRLDSCQALRQAKVKQLFNYIHECAQKGEAVDIGKAAFTTSLNLLSNLFFSVELANHKSSSSQEFKQLIWNIMEDIGKPNYADYFPVLKYVDPSGIRRRLASNFNKLIDVFQGFIRLRMSTNSSCGATNPNDVLDVLLNLYKGDDLNMDEINHLLVDIFDAGTDTTSSTFEWAMAELVKNPKMMKKAQAEIQQVLGKDSIIRESDIPNMPYLQAIIKETLRLHPPTVFLLPRKADADVELYGYVVPKNAQILVNLWALGRDPLVWKSPNVFKPERFLGSEIDFKGRDFGLLPFGAGRRICPGMNLAYRMLTLMLATLLQSFDWKVADGTNPQDMDMDEKFGIALQKTTPLQIIPVYKY |
|  | atggattatactactttagttatgattttgtctattgtgttcttttgttacaatctctttaatttgttgtttacaagaaagaatactaaattacctccaggtccaaagactattccaatttttggcaacatttttgaattaggtaagaaacctcatcagagctttgccaacttagctaaaattcacggcccacttatgtctttaaaattgggttctgttacgacaattgtcgtgtcctctgctgaagttgctagggagatgtttttgaaaaacgatcaattgttatctaacaggactgttccaaattctgttacagctggtgatcaccacaagaccaccatgtcttggttgccagtttctcaaaagtggaggaattttagaaaaattacagcagttcatttattatctccacaaagattagattcttgtcaagctttgagacaagccaaagttaaacaattattcaactacattcacgaatgtgctcaaaaaggcgaagctgtggatattggcaaagctgcttttactacatctttgaacttgttatcaaacctctttttcagcgttgaactggctaatcataaatcttctagctcacaggaatttaaacaactgatttggaacattatggaagatattggtaaacctaactacgctgactacttcccagttttaaaatacgttgatccatccggcatcagaagaagattagcttccaattttaacaaattgattgatgttttccagggtttcattagactaagaatgtctactaactcttcttgtggggcaactaatccaaatgacgtcttggatgttttgttgaatttgtataaaggtgatgatttgaatatggatgaaataaatcatctattggttgacatttttgacgcaggtactgatactacttcttccacttttgaatgggctatggccgagttggttaagaatccaaaaatgatgaagaaagcccaagctgaaattcaacaagtcttgggtaaggattctattattagagaatctgacattccaaacatgccatatttgcaagctattattaaagaaaccttgagattgcacccacctactgttttcttgttgccaagaaaggctgatgctgatgtagaattgtatggctacgttgttcccaaaaatgctcaaattttggttaacttgtgggctcttggtagagatccattagtgtggaagtctcctaatgtttttaaaccagaaagatttttaggttctgagatcgactttaaaggtagagattttggcttattgccatttggtgctggtaggagaatttgcccaggtatgaatttagcttatcgcatgctgactttaatgctagctactctattacaatcctttgattggaaggttgctgatggtactaacccacaagacatggacatggatgaaaaatttggcattgccttacaaaagactactccattgcaaattattccagtttataaatattaa |
| *PaTyH*  (AJD87467.1) | MDHTTLAMILSVIFLLYNLVKAIFSQSNTKLPPGPKPVPIFGNIFELGDKPHRSFANLAKIHGPLITLKLGSVTTIVVSSAEVAKEMFLTNDQLLANRNVPNSVTAGDHHKLTMSWLPVSPKWKTFRKITAVHLLSPQRLDACQALRHTKVKQLYEYVQECAKRGEAVDIGKAAFTTSLNLLSNLFFSVELANHTSSSSQEFKELIWDIMEDIGKPNYADYFPVLKCVDPWGIRRRLESNFDKLIEVFQSFIRKRLSTEPFSASAKTPNDVLDVLLNLLKEEELNMGEINHLLVDIFDAGTDTTSSTFEWAMAELVRNPEMMKKAQDEIEQVLGKDAIIQESDIPKMPYLQAIIKETLRLHPPTVFLLPRKASSNVELYGYVVPKNAQILVNLWAIGRDPTVWDNPNMFSPERFLNSDIDVKGRDFGLLPFGAGRRICPGMNLAYRMLTLMLATLLQSFDWKLGDGVNPKDLDMDEKFGIALQKTKPLQVIPVLKY |
|  | atggatcacactacattggcaatgattttgtccgttatcttcttgttgtacaatttagttaaagctatcttttctcaatctaatactaagttgccaccaggtccaaagccagttccaatttttggtaatatttttgaattgggtgataagccacatagatcttttgccaatctggccaagatccatggtccgttgattactcttaagttgggttcagttacaactattgttgtttcttctgctgaagttgctaaagaaatgttcttaactaatgatcaactattagctaatagaaatgttcccaactctgttacagctggtgatcatcataagttaactatgtcctggttaccagtttctccgaagtggaaaacttttagaaaaattactgcagtacatttgttgtctccacaaagattggatgcttgtcaggctttgaggcataccaaagttaaacaattgtacgaatatgttcaagaatgcgcaaaaagaggtgaagcagtggacattggtaaagctgcttttacaacttctttgaatttgttgtccaatttattcttctctgttgaattagctaaccatacttcttcttcttcacaagagtttaaggaattaatttgggatattatggaagatattggtaagccaaattatgccgattattttccagtcttgaagtgtgttgatccatggggtattagaagacgtttggaatctaactttgataagttgattgaggtgtttcaatcttttattagaaagaggttgtctacagaaccattttctgcatcagctaaaactccaaatgatgtgttagatgtgttgttaaatttgttgaaggaagaagaattaaatatgggtgaaattaaccacttattggttgatattttcgacgccggcactgatacaacttcttcaacctttgagtgggctatggcagaattggttagaaatccggagatgatgaaaaaggctcaagatgagattgaacaagtcttaggtaaagatgcaattattcaagaatctgatatcccaaagatgccatacttacaagctatcattaaggaaaccttgagattgcacccaccaacagttttccttttgccaagaaaagcatcttctaacgttgaactatatggttatgtcgttcccaaaaacgctcaaattttagttaatttatgggcaattggcagggatcctacagtttgggataatcctaacatgttctccccagagagatttttaaattctgatattgatgttaaagggagggattttggattgttgccatttggtgctgggagacgtatctgtccaggtatgaatttggcatacagaatgttgactttaatgttagcaaccttgttgcaatcctttgattggaagttgggtgatggtgttaacccgaaggatttggatatggatgagaaatttggtattgctctgcagaaaactaaacctcttcaagtcatccccgttttgaaatattaa |
| *OfTyH*  (AJD87464.1) | MDTPTLSYFISAITFYYIAFQIVKLGFNVIMTSKKTKRRRLPLPPGPKPLPIIGNVFELGPKPHRSFASLAKVYGPLMSLRLGSVTTIIVSSSDVAKEMFLKNDQPLSSTRTIPNSVTAGDHHKLTMSWLPVSPKWRSFRKITTFHLLSPQRLDACSGLRQAKVQQLYEYVLECSRTGQAVDIGKAAFTTSLNLLSKLFFSLELANHTSDKSQEFKELIWNIMEDIGKPNYADYFPCLKYFDPSGIRRRLACSFEKLIEVFQVIIRQRLSLSSSGTNDHNNDVLDVLLDLYQQKELSMEEINHLLVDIFDAGTDTTSSTFEWAMAELIKNPRMMETAQAEIKLILGKDLHIQESDIPKLPYLRAIIKETLRLHPPTVFLLPRKADADVELYGYTVPKNAQILVNLWALGRDPKVWENPDVFSPERFLGCDIDVKGRNFGLLPFGAGRRICPGMNLAYRMLTLMLATLLQSFDWKLPNEMNPQNLDMDEKFGIALQKTKPLQIIPLSKD |
|  | atggatactccaactttgtcttattttatttctgccatcacattctattacattgcatttcaaattgttaaattgggttttaacgttattatgacctcgaagaagactaaaagaagaagattaccattgccacctggcccaaaacccttgccaattattggtaatgttttcgaattaggtccaaagccacatagatcttttgcatcattggccaaagtctacggtccattgatgtctttgagattaggttcagtcactaccattattgtctcttcttcagatgtggctaaggaaatgtttttgaaaaatgatcaacccttgtcttccacaagaactattccaaattccgttactgctggtgatcatcataaattgactatgagctggttacccgtctctccaaaatggagatctttcaggaaaattacaactttccatttattatccccacaaaggttggatgcttgttctggtttaagacaagcgaaagtgcaacaattatatgaatatgttttagagtgctcaagaacaggtcaagctgttgatattggtaaagctgctttcactacttcattgaatttattgtctaagttgtttttctcattggaattggctaaccatacttctgataagtcacaagaattcaaggaattgatttggaatattatggaagacatcggtaagccaaactacgctgattactttccatgtttgaagtatttcgatccatctggtattaggcgccgtttagcttgttcttttgaaaaattgattgaagtttttcaggtcattattagacaaaggttatccctctcttcttctggtactaacgatcataataatgatgttttagatgttttgttagacttatatcaacagaaagaattgtctatggaggaaatcaatcatttgttagttgatatttttgacgctggtactgatactacttctagtacctttgaatgggcgatggccgaattaattaaaaacccaagaatgatggaaactgctcaggccgaaattaaattaattttgggtaaagatttacatattcaagaatctgacatcccgaaactgccatacttgagagctattattaaagaaactttgcgcttgcatccaccaactgtctttttgttgccaaggaaagctgacgctgatgttgaattgtatggctatactgtcccaaagaacgctcaaattttggttaatttatgggccttgggtagggatccaaaagtttgggaaaatccagatgttttctcaccagagagatttttgggttgtgacattgatgttaagggtaggaattttggtttattaccatttggtgctggtcgaagaatttgtccaggtatgaatttggcttataggatgctaactttaatgttggcaacattgttgcaatcttttgattggaaattaccaaatgaaatgaacccacaaaatttagatatggacgaaaagttcggcattgctttacaaaagactaaaccgttgcagattattcctttgtctaaagattaa |
| *AnTyH*  (AKI33952.1) | MDQTTLAMLLSALYLLYNLYKVIFTQSNSKLPPGPKPLPIFGNISELGAKPHRSFANLAKIHGPLITLKLGSVTTIVVSSAKVAEEMFLKNDLPLANRNVPNSVTAGDHHKLTMSWLPVSPKWKTFRKITAVHLLSPQRLDACQALRHAKVKQLYEYVYDCAKKGEAVDIGKAAFTTSLNLLSNLFFSVELAQHTSTSSQHFKQLIWDIMEDIGKPNYADYFPALKCVDPWGIRRRLAANFERLIDVFQDFIRPRLSMNPSSVTSASDVLDVLLNLYKEKELNMGEVNHLLVDIFDAGTDTTSSTFEWAMAELVRHPETMKKAQDEIEQVLGKDATIQEADIPKMPYLQAIIKETLRLHPPTVFLLPRKAATNVELYGYVVPKDAQILVNLWAIGRDPLVWDQPNVFSPERFLNSDVDVKGRDFGLLPFGAGRRICPGMNLAYRMLTLMLATLLQSFEWKVENGEKAEDLDMDEKFGIALQKTKPLQIIPVLKYC |
|  | atggatcaaactactttggctatgttgttgtctgccctctacttgttgtataacttgtataaagttattttcacccagagcaactccaaattgccaccaggtccaaaacctttaccaatttttggtaatatctctgaattaggtgctaagccccatagatcttttgctaatttagctaaaatccatggcccgttgattactttgaaattgggatctgtgaccacgatcgtggtttcttcagctaaagtggccgaagaaatgttcctgaagaatgatttaccattagctaataggaacgttccaaattctgttactgctggtgaccaccataaattaactatgtcttggttaccagtctctccaaagtggaaaacttttagaaaaattaccgccgtgcatttgttatcaccacaaagattagatgcttgtcaagctttgaggcacgcaaaagtaaaacaattgtatgaatatgtgtacgattgcgctaaaaagggtgaagctgttgatattggtaaagcagctttcactacatcgttgaatttattatctaatttgttcttctcagttgaattggctcaacatacttcgacttcttcccaacattttaaacaattgatttgggacattatggaagatattggtaagccaaactacgctgactactttccagctttgaaatgtgtcgatccatggggtattcgtagaagattggctgctaattttgaaaggttgattgacgtttttcaagattttattagaccgagattgtcaatgaatccatcctccgtcacttctgcttctgatgttctcgacgttttgttaaatttgtataaagaaaaagagcttaatatgggcgaagttaatcacttgttagtcgatatttttgatgctggtaccgatactacttcttctacatttgaatgggctatggctgagttggtccgtcatccagaaactatgaagaaagctcaggatgagatcgaacaagttttgggtaaagatgccactattcaagaagctgatattccaaaaatgccctatctgcaggctattattaaagaaacattgagattgcacccaccaactgttttcttgttgccaagaaaagcagctaccaacgtcgaactgtatggttatgttgttcctaaggatgctcagatcttggttaatttgtgggctattggtagagatccattggtttgggatcaaccaaacgtcttctctccagaaagatttttgaattcagatgtggatgttaaaggtagagattttggtttgttaccttttggcgctggtagaagaatttgtccaggtatgaacctagcttaccggatgttgactttgatgttagccactttattgcaatcttttgaatggaaagttgaaaatggtgaaaaagccgaagatttggatatggatgagaaattcggtatcgctttacagaaaacaaaaccattacaaattattccagttttgaaatattgttaa |
| *AoTyH*  (AKI33950.1) | MDQTTLAMLLSALYLLYNLYKVIFTQSNSKLPPGPKPLPILGNIFEVGNKPHRSFANLAKIHGPLITLKLGSVTTIVVSSAKVAEEMFLKNDLPLANRNVPNSVTAGDHHKLTMSWLPVSPKWKTFRKITAVHLLSPQRLDACQALRHAKVKQLHEYVQDCAKKGQAVDIGKAAFTTSLNLLSNLFFSVELAQHTSSSSQHFKELIWDIMEDIGKPNYADYFPALKCVDPWGIRRRLAANFERLIDVFQGFIRQRLSINSSTVTSASDVLDVLLNLYKEKELNMGEINHLLVDIFDAGTDTTSSTFEWAMAELVRHPEIMKKAQDEIEQVLGKDAIIQEADIPKMPYLQAIIKETLRLHPPTVFLLPRKATTNVELYGYVVPKNAQILVNLWAIGRDPLVWDNPNKFSPERFLNSDIDVKGRDFGLLPFGAGRRICPGMNLAYRILTLMLATLLQSFEWMVENGENPEDLDMDEKFGIALQKTKPLEIIPVIKHR |
|  | atggatcaaactactttggccatgttgctttccgcgttgtatctattatacaacttgtacaaagtcattttcactcaaagcaattctaaattgccaccaggtccaaagccactacccatcttaggtaatatttttgaagttggcaataaaccccatcgctcctttgctaatttggctaagattcacggcccattgattacattgaaacttggttccgtcactacaatcgttgtttcttctgctaaagttgccgaagaaatgttcttaaagaatgatttaccattggctaatagaaacgttccaaattctgttacagccggtgatcatcataagttgactatgtcttggttaccagtttcccctaaatggaaaacatttagaaaaattactgccgttcacttgttgagcccacaaagattggatgcttgtcaagccttgagacatgctaaagttaaacaattgcatgaatatgtacaagattgtgccaagaaaggtcaggctgtagatattggtaaagctgcttttacaacctcattgaacttgctatctaatttatttttctctgttgaattagcccaacatacttcttcctcttctcagcattttaaagaattaatttgggatattatggaggatattggtaagccaaattacgctgattattttcccgctttaaaatgtgttgatccatggggtatcagaagaagacttgcagctaacttcgaaagactgattgacgtctttcaaggttttattcgccagcggttgtctattaatagctctactgttacctccgcttcagatgttttagatgtgttgctgaatttatataaggaaaaagaattgaatatgggtgaaataaatcacctattggttgatattttcgatgccggtactgatacgacatcttctacatttgaatgggctatggctgaattggttagacatccagaaattatgaagaaagcgcaagatgaaattgagcaagttttggggaaagacgctatcattcaagaagctgatattccgaagatgccctacttgcaagctattattaaagaaactttgcgattacatcctccaaccgtctttttgttgccaagaaaagctactaccaatgttgaattgtacggttatgttgttccaaagaatgctcagatcctggttaatttgtgggctattggtagagacccattggtatgggacaatcctaataaattttctccagagagattcttgaattctgatattgatgttaaaggtcgtgacttcggtttattaccatttggtgccggtagaagaatttgtccaggtatgaatttggcctacagaatcctgactttaatgttggcaactttactgcaatctttcgaatggatggttgaaaatggtgaaaacccagaagatttggatatggatgaaaagttcggcattgctttgcagaaaacaaaacctttggaaattatcccagttattaaacatagataa |
| *MmTyH1*  (AKI33948.1) | MDQTTLAMLLSALYLLYNLFKVIFTQSNSKLPPGPKPLPILGNIFELGDKPHRSFNNLAKIHGPLITLKLGSVTTIVVSSAKVAEEMFLKNDLPLANRNVPNSVTAGDHHKLTMSWLPVSPKWKTFRKITAVHLLSPQRLDACQALRHAKVKQLYQYVQDCAKKGEAVDIGKAAFTTSLNLLSNLFFSVELAQHTSSSSQHFKELIWDIMEDIGKPNYADYFPALKCVDPWGIRRRLAANFERLIQVFQNFIRQRLSTDPSSVTNASDVLDVLLNLYKEKELNMGEINHLLVDIFDAGTDTTSSTFEWAMAELVRHPEIMKKAQDEIEQVLGKDATIQEADIPKMPYLQAIIKETLRLHPPTVFLLPRKATTNVELYGYVVPKNAQILVNLWAIGRDPLVWDQPNKFSPERFLNSDIDVKGRDFGLLPFGAGRRICPGMNLAYRMLTLMLATLLQSFEWKVQNGEKPEDLDMDEKFGIALQKTKPLEIIPVLKYR |
|  | atggatcaaactactttggctatgttattatccgccctataccttttgtataatttgttcaaggtcatttttactcaatctaattctaaattgccgccaggtccaaaaccattgccaattttaggcaatatctttgaattgggtgacaaaccacatagatcttttaataatttggccaaaattcatggtccattaatcacattaaaattaggttctgttacaactattgttgtttcttctgctaaagttgcagaagaaatgtttttgaagaatgacttgcccttggctaatagaaatgtaccaaattctgttactgctggtgatcaccataaattgactatgtcttggttgcccgtttctccaaaatggaaaacttttagaaagattaccgctgtgcacttgttgtctccacaaagacttgatgcctgccaggccttgagacatgctaaggttaaacaactgtaccagtacgtgcaagattgtgctaagaaaggtgaagccgttgatattggtaaagctgcgttcaccacttccctcaatttattgtctaacttgttcttttctgtcgaactagctcaacatacttcttctagctcccaacattttaaagaattaatttgggatattatggaagatataggtaaaccaaattatgctgattattttcctgccttgaaatgtgttgatccatggggtattagaagaagattagctgctaactttgaaagattgatccaagtttttcaaaacttcattagacaaagattatctactgatccatcttccgtcacaaatgcctctgatgttctagacgttttattgaacttgtataaagaaaaagagttaaacatgggtgagattaatcatttgttagttgatattttcgatgccggtactgatactacatcttcaacttttgaatgggctatggctgaattagtgcgtcacccagaaattatgaagaaagctcaagatgaaattgaacaagttctcggtaaagatgccacaattcaagaagctgatattcccaaaatgccatatttgcaagctatcattaaagaaactttgagattgcatcccccaactgtcttcttgctaccaagaaaagccaccaccaatgttgaattgtatggttatgttgttcctaaaaatgcccagattttggttaatttatgggctattgggagggatccattagtttgggatcaaccaaataagttttctccagagcgctttttaaattctgacatcgatgtcaaaggccgtgattttggtttattgccatttggggctggtagaagaatttgtccaggtatgaacctagcttatagaatgctaaccttaatgttagctactttgttgcaatcatttgaatggaaggtgcaaaatggcgaaaaacctgaagatttggatatggacgaaaagtttggtattgctttgcagaaaacaaaacctttagaaattattccagttttgaaatatagataa |
| *EvTyH*  (AKI33945.1) | MDHTTLAMILSAIFLLYNLAKAIFSHSNTKLPPGPKPVPIFGNIFELGEKPHRSFANLAKIHGPLITLKLGSVTTIVVSSAEVAKEMFLKNDLPLANRNVPNSVTAGDHHKLTMSWLPVSPKWKTFRKITAVHLLSPQRLDSCQALRHTKVKQLHQYVQECAKRGEPVDIGKAAFTTSLNLLSNLFFSVELANHTSSSSQEFKELIWEIMEDIGKPNYADYFPILKCVDPWGIRRRLASNFDKLIEVFQGFIRKRLSTGSFSAITPNDVLDVLLNLLKEKELNMGEINHLLVDIFDAGTDTTSSTFEWAMAELVRNQEMMKKAQDEIEQVLGKDAIIQESDIPKMPYLQAIIKETLRLHPPTVFLLPRKATSNVELYGYVVPKNAQILVNLWAIGRDPKVWDNPNMFSPERFLNSEIDVKGRDFGLLPFGAGRRICPGMNLAYRMLTLMLATLLQSFDWKLGDGVNPKDLDMEEKFGIALQKTKPLQVIPVLKY |
|  | atggatcatactactttagctatgattttgtctgctattttcctgttatataatttagctaaagccatttttagtcattctaacactaaacttccaccaggtccaaagcccgtccccatttttggtaatatttttgaattgggtgaaaaaccccatcgatcattcgctaatttggctaagatccacggtccactaattactttgaagttgggttctgtcaccactattgttgtatcttctgcagaagtcgcaaaggaaatgtttttgaaaaatgatttgccattggccaacaggaatgttccaaatagtgttactgccggtgatcaccacaaactaactatgtcatggttaccagtttctcccaaatggaaaacatttagaaaaataaccgctgtccatttattgtctccacaaagattggattcttgccaagctttaaggcatacaaaagttaaacaattacaccagtacgttcaagaatgtgctaaaagaggtgaaccagttgacattggtaaagctgcttttacaacctccttgaatttattatccaatttgttcttttccgttgaattagccaatcatactagttcatcttctcaggaatttaaagaattgatttgggaaattatggaagacattggaaaacccaattatgctgattattttccaatcctgaagtgcgttgatccatggggcattaggagaagattggcttcaaactttgataagttaattgaagtctttcaaggttttattcgcaagcgtttgtctactggttctttttctgccattactccaaacgatgttttagatgtgttattgaatttgttgaaagaaaaggaattaaatatgggtgaaattaaccatttgttagttgatatttttgatgctggtactgatactacttcatctactttcgaatgggccatggctgaattagttaggaaccaagaaatgatgaaaaaggctcaagatgagattgagcaggttttaggtaaggacgctatcatccaagaatctgatattccaaaaatgccatatttgcaagcaatcattaaagaaaccttgagattacacccgccaacagttttcttattaccaagaaaagctacctctaacgttgaattatatggttatgttgttccaaagaacgctcaaattttagttaatttgtgggctatcggtagagatccaaaagtttgggataatccaaatatgttctctccagaaaggtttttgaattctgaaatcgacgttaagggtagagattttggtttgttaccatttggtgcaggtagaagaatttgtccaggtatgaatttggcttataggatgttgacacttatgctagctacattgttacagtcttttgattggaagttaggcgatggtgtcaatccaaaagatctggatatggaagaaaagtttggtattgctttacaaaagactaaacctctgcaagttattccagtgttaaaatattaa |
| *PdTyH*  (AKI33942.1) | MDHTTLAMILSAIFLLYNLVKLAIFSQSNTKLPPGPKPLPIFGNIFELGDKPHRSFANLAKIHGPLITLKLGSITTIVVSSAEVAKEMFLKNDQPLANRNVPNSVTAGDHHKLTMSWLPVSPKWKTFRKITAVHLLSPQRLDACQALRHTNVKQLHEYVQECAQRGQPVDIGKAAFTTSLNLLSNLFFSVELANHTSSNSQEFKELIWDIMEDIGKPNYADYFPVLKCVDPWGIRRRLESNFDKLIEVFQGFIRKRLSTGSFSASETTPNDVLDVLLNLFKEKELNMGEINHLLVDIFDAGTDTTSSTFEWAMAELVRNPDMMKKAQDEIEQVLGRDAIIQESDIPKMPYLQAIIKETLRLHPPTVFLLPRKATTNVDLYGYVVPKNAQILVNLWAIGRDPTVWDNPNMFSPERFLNSDIDVKGRDFGLLPFGAGRRICPGMNLAYRMLTLMLATLLQSFNWKLGDGVNPKDLDMDEKFGIALQKTKPLQVIPVFKY |
|  | atggatcatacaacattggctatgatcttatccgctatatttttgttgtacaatttggttaaattagctatttttagccaatcaaatactaaattgccaccaggtccaaagcctttgccaatttttggtaatatttttgaattgggcgataaaccacacagatcttttgctaatctagcaaaaattcatggtccattgattacattgaagttgggttctattacaactattgttgtgtcttccgctgaagttgctaaggagatgtttttgaaaaatgatcaaccattagctaacaggaatgtgccaaattctgttactgcgggtgatcaccacaaattgactatgtcctggttgccagtttctcccaaatggaaaacatttagaaaaattaccgccgttcatttgttaagtccacagagactagatgcctgtcaagctttgagacacaccaatgtcaagcaactccatgaatatgttcaagaatgtgctcaaagaggtcaaccagttgatattggtaaagctgcctttaccaccagcttaaatttgttgtcaaatttatttttctccgtcgagttggctaatcatacttcctctaactcccaagaatttaaagaattaatttgggatattatggaagatattggcaaaccaaattatgctgattacttcccagtgttaaaatgtgttgatccatggggtattcgtagacggttagaatccaacttcgataagttaattgaagtttttcaaggctttatccgtaagcgtttgtctactggttctttttcagcttctgaaaccacccctaatgatgttttggatgttttactgaatttgttcaaagaaaaggaattgaacatgggagaaattaaccacttgttagttgatatttttgatgcaggtaccgatactacatcttccacttttgaatgggctatggctgagttggtccgtaatccagatatgatgaagaaagctcaggatgaaattgaacaagttttgggtagagatgccattatccaagaatcagatattccaaagatgccatatctgcaagctatcattaaggaaactttgaggttacatccaccaactgttttcttactgcctagaaaagctactactaatgttgacctttatggctacgttgttcctaaaaatgctcaaattttggttaatttgtgggctatcggcagggacccaacagtttgggataatccaaacatgttttctccagagagatttttaaattctgatatagatgtcaagggccgggattttggtttattaccatttggtgctggtcggcgcatttgtccaggtatgaatttggcttacaggatgttgactctaatgttagctactttattgcaatcttttaattggaaattgggtgatggtgttaacccaaaggatttggatatggatgaaaaattcggtatcgctttgcaaaagacaaaacctttacaagtgattcccgtctttaagtactaa |
| *MmTyH2*  (AKI33937.1) | MDFLTLVMILSMIFFFYNLLKMIFTTHSDAQLPPGPKPMPIFGNIFELGEKPHRSFANLAKTHGPLMSLRLGRVTTIVVSSAEVAKEMFLKNDQSLADRSVPNSVTAGDHHKLTMSWLPVSPKWKNFRKITAVHLLSPQRLDACNALRHAKVKQLYEYVQECALKGEAVDIGKAAFTTSLNLLSNLFFSVELANHTSNTSQEFKQLIWDIMEDIGKPNYADYFPLLKYVDPSGIRRRLAANFDKLIDVFQSFICKRLSSAYSSATSLDDVLDVLLKLYKEKELNMGEINHLLVDIFDAGTDTTSNTFEWAMSELIRNPTMMKRAQNEIALVLGKDNGTIQESDIANMPYLQAIIKETLRLHPPTVFLLPRKAITYVKLYGYIVPKNAQILVNLWAIGRDPKVWKNPNEFLPDRFLNSDIDVKGRDFGLLPFGAGRRICPGMNLAYRMLTLMLATLLQSFDWKLPHGNSPMDLDMDEKFGIALQKTKPLEIIPVIKY |
|  | atggactttttgactttagttatgattttatctatgattttcttcttttataacttgttaaaaatgatttttactacccattctgatgctcaattgccaccagggccaaaaccaatgccaatttttggtaatatttttgagttgggcgagaaaccacatagatcttttgccaatctagctaagacccatggtccattgatgtctttgaggttaggtcgtgttactactattgttgtttctagcgctgaagttgctaaagaaatgtttttgaaaaatgatcaatctttggcggacaggtctgttccaaattcagtaactgccggggaccatcacaaattgacaatgtcttggttacccgtttcacccaaatggaagaactttagaaaaattaccgctgttcatttgttgtctccacaaagattggacgcttgtaacgctttgagacatgccaaagttaaacaattgtatgaatacgttcaagaatgtgccttaaaaggtgaagctgttgatattggcaaagccgcattcaccacttcgttgaatttattgtctaatttgttcttttctgttgaacttgctaaccacacttcgaatacgtcgcaagaatttaaacaattgatttgggatattatggaggatattggtaaaccaaactacgccgattattttcctttgttgaaatacgttgatccaagcggtattagaagaagattagccgccaattttgataagttaattgatgtttttcagtcttttatctgcaaacgtttgtcttctgcttattcctccgctacttccttggatgacgttttggatgttttgttaaaattgtataaagaaaaggaactcaatatgggtgaaattaatcatttattggtcgatatttttgacgctggtaccgatacaacttctaatacttttgaatgggcaatgtctgaattgattagaaatcccaccatgatgaagagagcacagaatgaaatcgctttagttctaggtaaagataacggtaccattcaagaatctgatatcgccaacatgccatatcttcaagctatcattaaagaaacactgagattacatccacctactgtatttttattgccaaggaaagctattacctatgttaaattatatggctatattgttccaaagaatgcccaaatcttggttaatttatgggctattggtagggatcccaaagtatggaaaaatccaaatgaatttctgcctgatagatttttgaattctgacattgacgttaaaggtagagattttggtttgttaccctttggtgctggtagaagaatttgtccaggcatgaatttagcttatagaatgttaactttaatgttagctaccttgttgcaatcttttgattggaaattgccccatggcaattcaccaatggatttagatatggacgaaaaattcggtattgctttacagaaaacgaagccattggaaatcattccagttattaaatattaa |
| *DbB5GT*  (CAB56231.1) | MGTHSTAPDLHVVFFPFLAHGHMIPSLDIAKLFAARGVKTTIITTPLNASMFTKAIEKTRKNTETQMEIEVFSFPSEEAGLPLGCENLEQAMAIGANNEFFNAANLLKEQLENFLVKTRPNCLVADMFFTWAADSTAKFNIPTLVFHGFSFFAQCAKEVMWRYKPYKAVSSDTEVFSLPFLPHEVKMTRLQVPESMRKGEETHFTKRTERIRELERKSYGVIVNSFYELEPDYADFLRKELGRRAWHIGPVSLCNRSIEDKAQRGRQTSIDEDECLKWLNSKKPDSVIYICFGSTGHLIAPQLHEIATALEASGQDFIWAVRGDHGQGNSEEWLPPGYEHRLQGKGLIIRGWAPQVLILEHEATGGFLTHCGWNSALEGISAGVPMVTWPTFAEQFHNEQLLTQILKVGVAVGSKKWTLKPSIEDVIKAEDIEKAVREVMVGEEGEERRRRAKKLKEMAWRAIEEGGSSYSDLSALIEELKGYHTSEKE |
|  | atgggtactcattctactgctccagacttgcatgttgttttttttccatttttggcccacggtcatatgatcccatctttggatattgctaagttgtttgctgctagaggtgttaagactaccattattactactccattgaacgcctctatgttcaccaaagctattgaaaagactaggaagaacactgaaacccagatggaaatcgaagttttctcattcccatctgaagaagctggtttgccattgggttgtgaaaatttggaacaagctatggctattggtgccaacaatgaatttttcaacgctgccaacttgctgaaagaacagttggaaaatttcttggtcaagaccagaccaaattgcttggttgctgatatgttttttacttgggctgctgattctaccgccaagtttaacattccaactttggttttccacggcttttctttctttgctcaatgcgctaaagaagtcatgtggcgttacaaaccttacaaggctgtttcttctgataccgaggttttttctttgccattcttgccacatgaagtcaagatgactagattgcaagttccagaatctatgaggaagggtgaagaaactcatttcactaagagaaccgaaaggatcagagaattggagagaaaatcttacggtgtgatcgtcaactctttctacgaattggaaccagattacgccgacttcttgagaaaagaattgggtagacgtgcttggcatattggtccagtttctttgtgtaacagatccattgaagataaggcccaaagaggtagacaaacctctattgatgaagatgaatgcctgaagtggctgaattctaaaaagccagattccgttatctacatctgctttggttctaccggtcatttgattgctccacaattgcacgaaattgctactgctttggaagcttctggtcaagatttcatttgggctgttagaggtgatcatggtcaaggtaattctgaagaatggttgccaccaggttacgaacatagattacaaggtaagggtttgatcattagaggttgggctccacaagttttgattttggaacatgaagctactggtggtttcttgactcattgtggttggaattctgcattggaaggtatttctgctggtgttccaatggttacttggccaacttttgctgaacaattccataacgaacagttgttgacccaaatcttgaaagttggtgttgccgttggttctaagaaatggactttgaagccatccatcgaagatgttattaaggccgaagatatcgaaaaggccgttagagaagttatggttggagaagaaggtgaagaacgtagaagaagggctaaaaagttgaaagaaatggcttggagagctattgaggaaggtggttcttcttattctgatttgtccgccttgatcgaagaattgaaaggttaccatacctccgagaaagagtga |
| *UGT73DN1*  (Original: XP_010681315.1  Updated: KMT08362.1) | MGAEPQRLHVVFFPLMAAGHLIPTLDIAKLFAAHHVKTTIITTPLNAPCFTKPLESYKNLGHRIDIEIIPFPSKEAGLPEGLENFDQFTSDQMAVKFLKATELLQESFEKFLEKHKPNCIVTDMLMPFTNNVAAKFNIPRIVFHGCSYFALCMMHTLLKYQPHKSLLSDDEEFLVPNLPHEINLTRSRLPDMMRGQGDKELNDAWMKIFIHAMEAEENSFGVIMNSFYELEPEYVEYYRNVMGRKAWHIGPVSLCNRENEAKFQRGKDSSINEHECLKWLDSKKPKSVVYICFGSLAEVPTLQLREIAMGLEASEQDFIWVVRRGKENVEEEKIEEWLPYDFEDRMEGKGLIIRGWAPQVLILDHEAIGAFVTHCGWNSTLEGISCGVPMVTWPVFAEQFYNEKLVTEVLKTGVAVGAKKWSRILEVNLKSEDIKNAIRRVMVGEEALVLRSKAKKLKELARKAVEIGGSSYSDMHSLIQDLSSYNANGYKQYL |
|  | atgggtgctgaaccacaaagattgcacgttgttttttttccattgatggctgccggtcatttgattccaactttggatattgctaagttgttcgctgctcatcatgttaagactaccattattactaccccattgaacgctccatgtttcacaaaaccattggagtcttacaagaacttgggtcacagaattgacatcgagattatcccatttccatccaaagaagctggtttgccagaaggtttggaaaactttgatcaattcacctccgatcaaatggccgttaagtttttgaaggctactgagttgttgcaagagtcctttgaaaagttcttggaaaagcacaagccaaactgtatcgttaccgatatgttgatgccattcactaacaatgttgctgccaagttcaacatcccaagaatcgtttttcatggctgttcttacttcgctttgtgtatgatgcataccttgttgaaataccagccacacaagtctttgttgtccgatgatgaagaatttctggttccaaatttgccacacgaaatcaacttgaccagatctagattgccagatatgatgagaggtcaaggtgacaaagaattgaatgatgcttggatgaagattttcattcatgccatggaagccgaagaaaactctttcggtgttatcatgaacagcttctacgaattggaacctgaatacgttgagtactacagaaacgttatgggtagaaaggcttggcatattggtccagtttctttgtgtaacagagaaaacgaagccaagttccaaagaggtaaggattcctctattaacgaacacgaatgtttgaagtggctggattctaaaaagccaaagtccgttgtttacatctgctttggttctttggctgaagttccaacattgcagttgagagaaattgctatgggtttagaagcttccgaacaagatttcatctgggttgttagaaggggtaaagaaaacgtcgaagaagaaaagattgaagagtggttgccatacgacttcgaagatagaatggaaggtaagggtttgattattagaggttgggctccacaggttttgatcttggatcatgaagctattggtgctttcgttactcattgtggttggaactctactttggaaggtatttcttgtggtgttcctatggttacttggccagtttttgctgaacaattctacaacgaaaagttggtcaccgaagttttgaaaactggtgttgctgttggtgctaaaaagtggtctagaatcttggaagtcaacttgaagtccgaggatattaagaacgccattagaagagttatggttggtgaagaagctttggtcttgagatctaaagccaagaagttgaaagaattggctagaaaggcagttgaaatcggtggttcttcttactctgatatgcactccttgattcaggacttgtcatcttacaatgctaacggttacaagcagtacctgtga |
| *UGT73A36*  (Originally: XP_010691725.1  Updated: KMT01176.1) | MDDKSQQLHIVLFPFMAHGHMIPTLDIARLFAARGVKTTLITTPRNAPTFLTAIEKGNKSGAPTINVEVFNFQAQSFGLPEGCENLEQALGPGIRDRFFKAAAMLRDQLEHFLEKTRPNCLVADMFFPWATDSAAKFNIPRLVFHGHCLFALCALEIIRLHEPYNNASSDEEPFLLPHLPHEIELTRLQFSEELWKNGGDSDYKERSKAIKESELKCYGVLVNSFYELEPDYAEYFRKDLGRRAWNIGPVSLYNRSNEEKAQRGKQASIDEHECLKWLNSKKPNSVIYICFGSTMHMIPSQLNEIAMGLEASGKDFIWVVRNEDDLGEFEHRMEGKGLIIRGWAPQVLILEHEVIGAFVTHCGWNSTIEGIAAGVPMVTWPVFAEQFLNEKLITRVLRIGIPVGAKKWDCKPCEEYVVKKNDIEKALREVMEGNEAEERRTRAKEYKEMAWKALQEGGSSYSDLSALIDELRGLST |
|  | atggacgacaagtcccaacaattgcacatagttttgtttccattcatggcccatggtcatatgattccaactttggatattgctaggttgtttgctgctagaggtgttaagactactttgattactactccaagaaacgctccaactttcttgactgctattgaaaagggtaacaaatctggtgctccaaccattaacgttgaggtttttaacttccaagctcagtcttttggtttgcctgaaggttgtgaaaatttggaacaagctttaggtccaggtatcagagatagatttttcaaagctgctgccatgttgagagatcaattggaacatttcttggaaaagaccagaccaaattgcttggttgctgatatgttttttccatgggctactgattctgctgccaagttcaatattccaagattggtttttcatggccattgcttgttcgctttgtgtgctttggaaatcatcagattgcatgaaccttacaacaacgcctcttctgatgaagaaccatttttgttaccacacttgccacacgaaattgaattgaccagattgcagttctctgaagagttgtggaagaatggtggtgattccgattacaaagaaagatccaaggccatcaaagaatccgaattgaaatgttacggtgtcctggttaactccttctatgaattggaaccagattacgccgaatacttcagaaaagatttgggtagacgtgcatggaacattggtccagtttcattatacaacaggtccaacgaagaaaaggctcaaagaggtaaacaagcctccattgatgaacatgaatgtctgaagtggctgaactctaagaagccaaattccgttatctacatctgtttcggttctaccatgcatatgatcccatctcaattgaacgaaatcgctatgggtttagaagcttccggtaaagatttcatctgggttgttagaaacgaagatgacttgggtgaattcgaacatagaatggaaggtaagggtttgattattagaggttgggctccacaagtcttgatcttggaacatgaagttattggtgctttcgttactcattgtggttggaactctaccattgaaggtattgctgctggtgttccaatggttacttggccagtttttgctgaacaattcttgaacgagaagttgatcaccagagttttgagaattggtattccagttggtgctaaaaagtgggattgcaaaccatgtgaagaatacgttgttaagaagaacgatatcgaaaaggccttgagagaagttatggaaggcaatgaagctgaagaaagaagaactagagccaaagagtacaaagagatggcttggaaggctttacaagaaggtggttcatcttactctgatttgtccgctttgatcgatgaattgagaggtttgtctacctaa |
| *UGT73A38*  (Original: XP_010691729.1  Updated: KMT01178.1) | MSAEPQDFHVVLFPFMAHGHMIPMLDIARLFTARDNVKATIITTQLNIATITKDIESNKTTRTPIFNIELFKFPAQEVGLPEGFENLEQVLRPEFFDKFFEATELLNEQLEKYLEKVRPNCLVADMFFPWATDSAAKFNIPRLVFHGTSIIALCAEEIIRLYEPFNNVSSDEEPFVLPLLPHEIQLRRSQISEGLTENEETDYKRKIANIKESELKSYGVIINSFYEMEPDYAKFYSKELRRRSWNVGPVSLSNRSIEDKNQGDKQALIDEHECLQWLNSKKPDSVTYICFGSTTSFIAPQLHEIAMALEASGEDFIWVVKDDENVKGEEWLPTGFKQRMEGKGFIITGWAPQVLILEHEAIRAFMTHCGWNSILEGISAGVPMVTWPIFAEQFYNEKLVNQILKTSVPIGAKKWSRRHSLEDLIKCDAIEKALKEIMVGKEAEERRTRAKNLKKKAYMAVEEGGSSYSDLTALINELRGYRLQEQE |
|  | atgtccgctgaaccacaagatttccacgttgttttgtttccattcatggctcatggtcatatgatcccaatgttggatattgctaggttgttcactgctagagataacgttaaggctaccattatcactacccaattgaacattgccaccatcaccaaggatatcgaatctaacaaaactactaggacgcccattttcaacatcgagttgtttaagtttccagctcaagaagttggtttgccagaaggttttgaaaacttggaacaagttttgaggccagagttcttcgacaagttttttgaagctactgagttgttgaacgagcagttggaaaagtacttggaaaaggttagaccaaactgcttggttgctgatatgttttttccatgggctactgattctgctgccaagtttaacattccaagattggtttttcatggcacctccattattgctttgtgtgccgaagaaattatcaggttgtatgaacccttcaacaacgtttcctctgatgaagaaccattcgttttgccattactgccacacgaaattcagttgagaagatcccaaatttctgaaggtttgaccgaaaacgaagaaaccgattacaagagaaagatcgccaacatcaaagagtccgaattgaaatcttacggcgtcatcatcaacagcttctacgaaatggaaccagattacgctaagttctactccaaagaattgcgtagaagatcttggaacgttggtccagtttctttgtccaacagatccatcgaagataagaatcaaggtgataagcaagccttgatcgatgaacatgaatgcttgcaatggctgaattctaagaagccagattccgttacctacatttgtttcggttctaccacttctttcattgctccacaattgcacgaaattgctatggctttggaagcttctggtgaagatttcatttgggttgttaaggatgacgaaaacgtcaaaggtgaagaatggttgccaactggttttaagcaaagaatggaaggtaagggtttcattattactggttgggctccacaagtcttgattttggaacatgaagctattagagccttcatgactcattgtggttggaactctattttggaaggtatttctgctggtgttccaatggttacttggccaatttttgctgaacagttctacaacgagaaactggtcaaccagatcttgaaaacctctgttccaattggtgctaaaaagtggtctagaaggcattccttggaagatttgattaagtgtgatgccattgagaaggccctgaaagaaatcatggttggtaaagaagctgaagaaagaagaaccagagctaagaacttgaagaaaaaggcttacatggctgttgaagaaggtggttcatcttactctgatttgaccgctttgatcaacgaattgagaggttacagattgcaagagcaagagtga |
| *UGT73A39*  (Originally: XP_010691730.1  Updated: KMT01177.1) | MGAEEFHVVLFPFMAHGHMIPILDIARLFAVRDNVKATIITTPLNVTNFTKDNESNMESSAPIVNIEVFRFPAQEVGLPEGFENLEKVMKPELLNKFFKAAGMLNEQLEQYLEKIRPNCLVADMFFPWATESAAKFNIPRLVFHGTSNFALCAQEIVRLYKPFKNVTSDEEPFVLPSLPHDIQMRRLQIPEDLWKYDGTEFEKRMDSLKDSEIKSYGVLVNSFYELEPDYAEFFKKELGRKTWNIGPVSLSNRSVKDKSQREKEPLIDQHECLKWLNSKKPDSVVYICFGSTASFIAPQLHEMALALEVSGQEFIWIVKNDENGRSKEWLPPGFEQRTEGKGLIIRGWVPQVLILEHEAIGAFVTHCGWNSTLEGISAGVPMVTWPIFAEQFYNEKLVNQILNTGVPVGAKKWSRTPSIEDIIKMEAIEEALTDIMVGTEAEERRTRAKKLKILAYKAVEEDGSSYSDLTALINELSANRLQEQE |
|  | atgggtgccgaagaattccacgttgttttgtttccattcatggctcatggtcatatgatcccaattttggatattgctaggttgttcgccgttagagataatgttaaggctaccattattacgaccccattgaatgttaccaacttcaccaaggataacgagtccaatatggaatcttctgctccaatcgttaacatcgaagtttttagattcccagctcaagaagttggtttgccagaaggttttgaaaacttggaaaaggttatgaagcccgagttgttgaacaagttttttaaggctgctggtatgttgaacgaacagttggaacaatacttggagaagattagaccaaactgcttggttgctgatatgttttttccatgggctactgaatctgctgccaagtttaacattccaagattggtttttcatggcacctctaactttgctttgtgtgctcaagaaatcgtcaggttgtacaagccttttaagaacgttacctctgacgaagaaccattcgttttgccatctttgccacatgatatccagatgagaagattgcaaatccctgaagatttgtggaagtacgatggtactgaattcgaaaagagaatggactccttgaaggactctgaaatcaaatcttacggtgtcttggtcaactccttctatgaattggaaccagattacgccgagttcttcaaaaaagaattgggtagaaagacctggaacatcggtccagtttctttgtctaatagatccgttaaggacaagtcccagagagaaaaagaacctttgattgatcaacacgagtgcttgaagtggttgaattctaaaaagccagattccgttgtctacatctgttttggttctaccgcttcttttattgctccacaattgcacgaaatggctttggctttggaagtttctggtcaagaattcatttggatcgtcaagaatgacgagaacggtagatctaaagaatggttgccacctggttttgaacaaagaactgaaggtaagggtttgatcattagaggttgggttccacaagtcttgatcttggaacatgaagctattggtgctttcgttactcattgtggttggaactctactttggaaggtatttctgctggtgttccaatggttacttggccaatttttgctgaacagttctacaacgagaaactggtcaatcagattttgaataccggtgttccagttggtgctaaaaaatggtctagaaccccatccattgaggacattattaagatggaagccattgaagaagccttgaccgatattatggttggtacagaagctgaagaaagaagaactagagccaagaagctaaagattttggcttacaaggctgtcgaagaagatggttcatcttactctgatttgaccgccttgattaacgaattgtctgctaacagattgcaagagcaagagtga |

Table S2: List of the BioBricks used in this study

| **BioBrick name** | **Fw-primer** | **Rv-primer** | **Template DNA** |
| --- | --- | --- | --- |
| BB8 (Ptef1<-) | PR-5 | PR-6 | CEN.PK113-5D genome |
| BB10 |  |  |  |
| BB0618 (Ptdh3) | PR-7570 | PR-7571 | CEN.PK113-5D genome |
| BB0622 (Ptef1) | PR-7578 | PR-7579 | CEN.PK113-5D genome |
| BB0623 (Tadh1) | PR-7580 | PR-7581 | CEN.PK113-5D genome |
| BB0624 (Tcyc1) | PR-7582 | PR-7583 | CEN.PK113-5D genome |
| BB0629 (CAN1-UP-A) | PR-7540 | PR-7596 | CEN.PK113-5D genome |
| BB0630 (CAN1-DW-E) | PR-7597 | PR-7545 | CEN.PK113-5D genome |
| BB4151 (MjDOD_Gene1) | PR-25066 | PR25067 | pCfB9177 |
| BB4273 (DbB5GT_U1) | PR-25382 | PR-25383 | Synthetic gene |
| BB4440 (TyrHW13L_Gene2) | PR-25064, PR-26838 | PR-25065, PR-26839 | pCfB9195 |
| BB4686 (BvDOD1_gene1) | PR-27250 | PR-27251 | Synthetic gene |
| BB4687 (BgDOD1_gene1) | PR-27252 | PR-27253 | Synthetic gene |
| BB4688 (PgDOD_gene1) | PR-27254 | PR-27255 | Synthetic gene |
| BB4689 (SoDOD_gene1) | PR-27256 | PR-27257 | Synthetic gene |
| BB4690 (AtDOD_gene1) | PR-27258 | PR-27259 | Synthetic gene |
| BB4691 (AhDOD_gene1) | PR-27260 | PR-27261 | Synthetic gene |
| BB4692 (PaDOD_gene1) | PR-27262 | PR-27263 | Synthetic gene |
| BB4693 (SsDOD_gene1) | PR-27264 | PR-27265 | Synthetic gene |
| BB4694 (BgDOD2_gene1) | PR-27266 | PR-27267 | Synthetic gene |
| BB4695 (BvDOD2_gene1) | PR-27268 | PR-27269 | Synthetic gene |
| BB4696 (BvDOD3_gene1) | PR-27270 | PR-27271 | Synthetic gene |
| BB4697 (AnTyH_gene2) | PR-27272 | PR-27273 | Synthetic gene |
| BB4698 (AoTyH_gene2) | PR-27274 | PR-27275 | Synthetic gene |
| BB4699 (BaTyH_gene2) | PR-27276 | PR-27277 | Synthetic gene |
| BB4700 (CbTyH_gene2) | PR-27278 | PR-27279 | Synthetic gene |
| BB4701 (EvTyH_gene2) | PR-27280 | PR-27281 | Synthetic gene |
| BB4702 (MmTyH1_gene2) | PR-27282 | PR-27283 | Synthetic gene |
| BB4703 (MmTyH2_gene2) | PR-27284 | PR-27285 | Synthetic gene |
| BB4704 (OfTyH_gene2) | PR-27286 | PR-27287 | Synthetic gene |
| BB4705 (PaTyH_gene2) | PR-27288 | PR-27289 | Synthetic gene |
| BB4706 (PdTyH_gene2) | PR-27290 | PR-27291 | Synthetic gene |
| BB4730 (MjDOD_gene1) | PR-25066 | PR-27394 | Synthetic gene |
| BB4731 (TyrH(W13L)_gene2) | PR-27395 | PR-27396 | Synthetic gene |
| BB4732 (C-KlURA3-E) | PR-27409 | PR-27410 | p0018 |
| BB4733 (A-Ptef1-BvDOD1-Tcyc1-B) | PR-7587 | PR-7588 | USER and Ligation of BB0622, BB0624, BB4686 |
| BB4733 (A-Ptef1-BvDOD1-Tcyc1-B) | PR-7587 | PR-7588 | USER and Ligation of BB0622, BB0624, BB4686 |
| BB4734 (A-Ptef1-BgDOD1-Tcyc1-B) | PR-7587 | PR-7588 | USER and Ligation of BB0622, BB0624, BB4687 |
| BB4734 (A-Ptef1-BgDOD1-Tcyc1-B) | PR-7587 | PR-7588 | USER and Ligation of BB0622, BB0624, BB4687 |
| BB4735 (A-Ptef1-PgDOD-Tcyc1-B) | PR-7587 | PR-7588 | USER and Ligation of BB0622, BB0624, BB4688 |
| BB4735 (A-Ptef1-PgDOD-Tcyc1-B) | PR-7587 | PR-7588 | USER and Ligation of BB0622, BB0624, BB4688 |
| BB4736 (A-Ptef1-SoDOD-Tcyc1-B) | PR-7587 | PR-7588 | USER and Ligation of BB0622, BB0624, BB4689 |
| BB4736 (A-Ptef1-SoDOD-Tcyc1-B) | PR-7587 | PR-7588 | USER and Ligation of BB0622, BB0624, BB4689 |
| BB4737 (A-Ptef1-AtDOD-Tcyc1-B) | PR-7587 | PR-7588 | USER and Ligation of BB0622, BB0624, BB4690 |
| BB4738 (A-Ptef1-AhDOD-Tcyc1-B) | PR-7587 | PR-7588 | USER and Ligation of BB0622, BB0624, BB4691 |
| BB4739 (A-Ptef1-PaDOD-Tcyc1-B) | PR-7587 | PR-7588 | USER and Ligation of BB0622, BB0624, BB4692 |
| BB4740 (A-Ptef1-SsDOD-Tcyc1-B) | PR-7587 | PR-7588 | USER and Ligation of BB0622, BB0624, BB4693 |
| BB4741 (A-Ptef1-BgDOD2-Tcyc1-B) | PR-7587 | PR-7588 | USER and Ligation of BB0622, BB0624, BB4694 |
| BB4741 (A-Ptef1-BgDOD2-Tcyc1-B) | PR-7587 | PR-7588 | USER and Ligation of BB0622, BB0624, BB4694 |
| BB4742 (A-Ptef1-BvDOD2-Tcyc1-B) | PR-7587 | PR-7588 | USER and Ligation of BB0622, BB0624, BB4695 |
| BB4742 (A-Ptef1-BvDOD2-Tcyc1-B) | PR-7587 | PR-7588 | USER and Ligation of BB0622, BB0624, BB4695 |
| BB4743 (A-Ptef1-BvDOD3-Tcyc1-B) | PR-7587 | PR-7588 | USER and Ligation of BB0622, BB0624, BB4696 |
| BB4743 (A-Ptef1-BvDOD3-Tcyc1-B) | PR-7587 | PR-7588 | USER and Ligation of BB0622, BB0624, BB4696 |
| BB4744 (B-Ptdh3-AnTyH-Tadh1-C) | PR-7589 | PR-7590 | USER and Ligation of BB0618, BB0623, BB4697 |
| BB4745 (B-Ptdh3-AoTyH-Tadh1-C) | PR-7589 | PR-7590 | USER and Ligation of BB0618, BB0623, BB4698 |
| BB4746 (B-Ptdh3-BaTyH-Tadh1-C) | PR-7589 | PR-7590 | USER and Ligation of BB0618, BB0623, BB4699 |
| BB4747 (B-Ptdh3-CbTyH-Tadh1-C) | PR-7589 | PR-7590 | USER and Ligation of BB0618, BB0623, BB4700 |
| BB4748 (B-Ptdh3-EvTyH-Tadh1-C) | PR-7589 | PR-7590 | USER and Ligation of BB0618, BB0623, BB4701 |
| BB4749 (B-Ptdh3-MmTyH1-Tadh1-C) | PR-7589 | PR-7590 | USER and Ligation of BB0618, BB0623, BB4702 |
| BB4750 (B-Ptdh3-MmTyH2-Tadh1-C) | PR-7589 | PR-7590 | USER and Ligation of BB0618, BB0623, BB4703 |
| BB4751 (B-Ptdh3-OfTyH-Tadh1-C) | PR-7589 | PR-7590 | USER and Ligation of BB0618, BB0623, BB4704 |
| BB4752 (B-Ptdh3-PaTyH-Tadh1-C) | PR-7589 | PR-7590 | USER and Ligation of BB0618, BB0623, BB4705 |
| BB4753 (B-Ptdh3-PdTyH-Tadh1-C) | PR-7589 | PR-7590 | USER and Ligation of BB0618, BB0623, BB4706 |
| BB4773 (A-Ptef1-MjDOD-Tcyc1-B) | PR-7587 | PR-7588 | USER and Ligation of BB0622, BB0624, BB4730 |
| BB4774 (B-Ptdh3-TyH(W13L)-Tadh1-C) | PR-7589 | PR-7590 | USER and Ligation of BB0618, BB0623, BB4731 |
| BB5051 (UGT73DN1_Gene1) | PR-27946 | PR-27947 | Synthetic gene |
| BB5052 (UGT73A36_Gene1) | PR-27948 | PR-27949 | Synthetic gene |
| BB5053 (UGT73A38_Gene1) | PR-27950 | PR-27951 | Synthetic gene |
| BB5054 (UGT73A39_Gene1) | PR-27952 | PR-27953 | Synthetic gene |
| BB5448 (UGT73A36_Gene1) | PR-27948 | PR-27949 | Synthetic gene |
| BB5638 (PgDOD*_full_U1) | PR-27255 | PR-27254 | Genomic DNA of Lib3-iso21 |
| BB5639 (PgDOD*_truncated_U1) | PR-29406 | PR-27254 | Genomic DNA of Lib3-iso21 |
| BB5651 (ScQDR1 gRNA-donor disruption cassette) | PR-29952 | PR-29953 | Pooled transporter library, Addgene accession numbers 153101 - 153106 |
| BB5652 (pTAJAK71 with Gibson overhangs compatible with fusion to BB5651) | PR-29954 | PR-29955 | pTAJAK71 |
| BB5653(ScQDR2 gRNA-donor disruption cassette) | PR-29956 | PR-29957 | Pooled transporter library, Addgene accession numbers 153101 - 153106 |
| BB5654 (pTAJAK71 with Gibson overhangs compatible with fusion to BB5653) | PR-29958 | PR-29959 | pTAJAK71 |
| BB5655(ScALP1 gRNA-donor disruption cassette) | PR-29960 | PR-29961 | Pooled transporter library, Addgene accession numbers 153101 - 153106 |
| BB5656 (pTAJAK71 with Gibson overhangs compatible with fusion to BB5655) | PR-29962 | PR-29963 | pTAJAK71 |
| BB5657(ScYJR015W1 gRNA-donor disruption cassette) | PR-29964 | PR-29965 | Pooled transporter library, Addgene accession numbers 153101 - 153106 |
| BB5658 (pTAJAK71 with Gibson overhangs compatible with fusion to BB5657) | PR-29966 | PR-29967 | pTAJAK71 |
| BB5753 (pET_Gibson) | PR-29886 | PR-29887 | pET-28a plasmid. Kindly provided by the Enzyme Engineering and Structural Biology group, DTU, Denmark. |
| BB5754 (BvSGT2_Gibson-pET) | PR-29884 | PR-29885 | Synthetic gene |

Table S3: List of Primers used in this study.

| **Primer** | **Sequence (5’-> 3’)** |
| --- | --- |
| PR-5 | ACCTGCACUTTGTAATTAAAACTTAG |
| PR-6 | CACGCGAUGCACACACCATAGCTTC |
| PR-225 | CTCCTTCCTTTTCGGTTAGAG |
| PR-339 | GCTCATTAGAAAGAAAGCATAGC |
| PR-7540 | CTCTATCAATGAAAATTTCGAGGA |
| PR-7545 | GCTAAAACTTTGATGGAAGCG |
| PR-7570 | ATCACTCGTACAGCGGTA ATAAAAAACACGCTTTTTCAGTTCG |
| PR-7571 | ACCTGCACU TTTGTTTGTTTATGTGTGTTTATTCGA |
| PR-7578 | ATCACTCGTACAGCGGTA GCACACACCATAGCTTC |
| PR-7579 | ACCTGCACU TTGTAATTAAAACTTAGATTAGATTG |
| PR-7580 | ATCGCACGU GCGAATTTCTTATGATTTATG |
| PR-7581 | GGCTGAGTACAACTAGCT GAGCGACCTCATGCTATA |
| PR-7582 | ATCGCACGU ATCCGCTCTAACCGAAAAG |
| PR-7583 | GGCTGAGTACAACTAGCT TTCTCAAGCAAGGTTTTC |
| PR-7587 | ACACACTGTATAGAGGGTTGTGGATACCGCTGCACGTACTAGGATAGCGTCTCACACCATATCACTCGTACAGCGGTA |
| PR-7588 | ATGTCTAGGATCGTAGGTACCCGGTCTTACAGAAATGGCGCGCTCACATGACGTTACATTGGCTGAGTACAACTAGCT |
| PR-7596 | ACACACTGTATAGAGGGTTGTGGATACCGCTGCACGTACTAGGATAGCGTCTCACACCATCTCGCCATTTACTCTCGT |
| PR-7597 | CATTGGGTGAAAGTGTCTCACGTTACATACTCAGCCATAGTAGTCCAGGAGCCCGATACTCATAGGTGATGAAGATGA |
| PR-24714 | GAACGAACGTCAAGAGAAGCAC |
| PR-25382 | AGTGCAGGU AAA ACA ATG GGT ACT CAT |
| PR-25383 | CGTGCGAU TCA CTC TTT CTC GGA GGT |
| PR-27250 | AGTGCAGGU AAAACA ATG GGT TCT GAA GAT AAT AT |
| PR-27251 | ACGTGCGAU TTA AGT AGA AGT AAA TTT ATA AGA AC |
| PR-27252 | AGTGCAGGU AAAACA ATG GGT GGT GAA AAG AA |
| PR-27253 | ACGTGCGAU TTA AGT AGA AGT AAA TTT AT |
| PR-27254 | AGTGCAGGU AAAACA ATG GGT GTA GGT AAA GAA G |
| PR-27255 | ACGTGCGAU TTA AAT AGA AGT AAA TTT ATA AGA AGC |
| PR-27256 | AGTGCAGGU AAAACA ATG ATT TTG CAT AAT TTT CAA C |
| PR-27257 | ACGTGCGAU TTA ATT TGG AGA AAA TTT ATA AGA ACC |
| PR-27258 | AGTGCAGGU AAAACA ATGGGTTCTCAAGAAATTA |
| PR-27259 | ACGTGCGAU TTA AGA AGA AAC AAA TTT ATA AGA ACC |
| PR-27260 | AGTGCAGGU AAAACA ATG GGT TCT CAA GAA ATT AT |
| PR-27261 | ACGTGCGAU TTA TGA AGA AAC AAA TTT ATA AGA ACC |
| PR-27262 | AGTGCAGGU AAAACA ATG GAT GTT AAA GAC ATG AT |
| PR-27263 | ACGTGCGAU TTA TGA AGA AGT AAA TTT ATA ACA AGC |
| PR-27264 | AGTGCAGGU AAAACA ATG GGT TCT AAT AAT AAT AAT G |
| PR-27265 | ACGTGCGAU TTA TGA AGA AGT GAA TTT ATA G |
| PR-27266 | AGTGCAGGU AAAACA ATG GGT GGT GAA AAG AAA |
| PR-27267 | ACGTGCGAU TTA AGT AGA AGT AAA TTT ATA AGA AG |
| PR-27268 | AGTGCAGGU AAAACA ATG AAA ATG ATG AAT GGT GA |
| PR-27269 | ACGTGCGAU TTA AGC AGA AGT AAA TTT ATA AG |
| PR-27270 | AGTGCAGGU AAAACA ATG AAA ATG ATG AAT GGT G |
| PR-27271 | ACGTGCGAU TTA AGC AGA GGT GAA TTT AT |
| PR-27395 | AGTGCAGGU AAAACA ATG GAT CAC GCA ACA TTA GCT AT |
| PR-27396 | ACGTGCGAU TTA GTA TCT TGG AAT TGG GAT CAA |
| PR-27272 | AGTGCAGGU AAAACA ATG GAT CAA ACT ACT TTG GC |
| PR-27273 | ACGTGCGAU TTA ACA ATA TTT CAA AAC TG |
| PR-27274 | AGTGCAGGU AAAACA ATG GAT CAA ACT ACT TTG GC |
| PR-27275 | ACGTGCGAU TTA TCT ATG TTT AAT AAC TG |
| PR-27276 | AGTGCAGGU AAAACA ATG GAT AAT ACT ACT TTG GC |
| PR-27277 | ACGTGCGAU TTA AAT TGA ATT TTT AAA AAT TGG |
| PR-27278 | AGTGCAGGU AAAACA ATG GAT TAT ACT ACT TTA GT |
| PR-27279 | ACGTGCGAU TTA ATA TTT ATA AAC TGG |
| PR-27280 | AGTGCAGGU AAAACA ATG GAT CAT ACT ACT TTA GC |
| PR-27281 | ACGTGCGAU TTA ATA TTT TAA CAC TGG A |
| PR-27282 | AGTGCAGGU AAAACA ATG GAT CAA ACT ACT TTG GC |
| PR-27283 | ACGTGCGAU TTA TCT ATA TTT CAA AAC TGG |
| PR-27284 | AGTGCAGGU AAAACA ATG GAC TTT TTG ACT TTA G |
| PR-27285 | ACGTGCGAU TTA ATA TTT AAT AAC TGG AAT G |
| PR-27286 | AGTGCAGGU AAAACA ATG GAT ACT CCA ACT TTG TC |
| PR-27287 | ACGTGCGAU TTA ATC TTT AGA CAA AGG A |
| PR-27288 | AGTGCAGGU AAAACA ATG GAT CAC ACT ACA TTG |
| PR-27289 | ACGTGCGAU TTA ATA TTT CAA AAC GGG G |
| PR-27290 | AGTGCAGGU AAAACA ATG GAT CAT ACA ACA TTG |
| PR-27291 | ACGTGCGAU TTA GTA CTT AAA GAC GGG AA |
| PR-27946 | AGTGCAGGU AAAACA ATG GGTGCTGAACCACAAAG |
| PR-27947 | CGTGCGAU TCACAGGTACTGCTTGTAA |
| PR-27948 | AGTGCAGGU AAAACA ATGGACGACAAGTCCCAACA |
| PR-27949 | CGTGCGAU TTAGGTAGACAAACCTCTCA |
| PR-27950 | AGTGCAGGU AAAACA ATGTCCGCTGAACCACAAGA |
| PR-27951 | CGTGCGAU TCACTCTTGCTCTTGCAATC |
| PR-27952 | AGTGCAGGU AAAACA ATGGGTGCCGAAGAATTCCACG |
| PR-27953 | CGTGCGAU TCACTCTTGCTCTTGCAATC |
| PR-28955 | GTACCTACGATCCTAGAC |
| PR-28956 | TAAACAAACAAAAGTGCAGG |
| PR-25066 | AGTGCAGGU AAAACA ATG AAG GGA ACC TAC TAC ATC AA |
| PR-25067 | CGTGCGAU TTA CGT CGG TCT TTT GGG TGG |
| PR-25064 | ATCTGTCAU AAAACA ATG GAT CAC GCA ACA TTA GCT AT |
| PR-25065 | CACGCGAU TTA GTA TCT TGG AAT TGG GAT CAA |
| PR-26838 | ACTCAAAGGU AGATGATGTAGTATCAGTACC |
| PR-26839 | ACCTTTGAGU GGGTTATGACTGAGTTAA |
| PR-27948 | AGTGCAGGU AAAACA ATGGACGACAAGTCCCAACA |
| PR-27949 | CGTGCGAU TTAGGTAGACAAACCTCTCA |
| PR-29406 | CGTGCGAU TCAATTCAGCTTTAGATTTC |
| PR-29952 | GCAGTGAAAGATAAATGATCATAGTAGATAGACCCTGCCA |
| PR-29953 | TAACTAATTACATGACTCGAATAAACCACAATAGTGACAT |
| PR-29954 | CACTATTGTGGTTTATTCGAGTCATGTAATTAGTTAATCG |
| PR-29955 | AGGGTCTATCTACTATGATCATTTATCTTTCACTGCGGAG |
| PR-29956 | GCAGTGAAAGATAAATGATCTAGCTCAGTGCGCTTTCACT |
| PR-29957 | AACTAATTACATGACTCGAGTCAATATCGAATTTTCTTTC |
| PR-29958 | GAAAATTCGATATTGACTCGAGTCATGTAATTAGTTAATCGCG |
| PR-29959 | AAAGCGCACTGAGCTAGATCATTTATCTTTCACTGCGGAG |
| PR-29960 | GCAGTGAAAGATAAATGATCAGTTAAAGCAGCGGCATATA |
| PR-29961 | ATTAACTAATTACATGACTCGAGCCGGCGTGTGCCAATGG |
| PR-29962 | TGGCACACGCCGGCTCGAGTCATGTAATTAGTTAATCGCG |
| PR-29963 | TGCCGCTGCTTTAACTGATCATTTATCTTTCACTGCGGAG |
| PR-29964 | GCAGTGAAAGATAAATGATCTTATCCTCGGAATTTGGCTC |
| PR-29965 | CGATTAACTAATTACATGACTCGAAAAAGAACTTTCTTCCTTTG |
| PR-29966 | GGAAGAAAGTTCTTTTTCGAGTCATGTAATTAGTTAATCGCGTG |
| PR-29967 | AAATTCCGAGGATAAGATCATTTATCTTTCACTGCGGAG |
| PR-29884 | CTTTATTTTCAGGGCCATAAAACAATGGACGACAAG |
| PR-29885 | GTGGTGGTGGTGCTCGAGTTAGGTAGACAAAC |
| PR-29886 | ATGGCCCTGAAAATAAAG |
| PR-29887 | CTCGAGCACCACCACCAC |

Table S4: List of plasmids used in this study

| **Plasmid** | **Parent Vector** | **BioBricks** | **Source** |
| --- | --- | --- | --- |
| **Episomal Plasmids** |  |  |  |
| pCfB2312(CEN/ARS_Cas9_kanMX) |  |  | [1] |
| pCfB11487 (pET-BvSGT2) |  | BB5753, BB5754 |  |
| **gRNA Plasmids** |  |  |  |
| pTAJAK-71 (pESC-NatMXsyn-USER) |  |  | [2] |
| pCfB2310 (SNR52p-gRNA.CAN1-SUP4t_natMX) |  |  | [2] |
| pCfB3020 (p-gRNA-X-2) |  |  | [2] |
| pCfB3041 (p-gRNA X-3) |  |  | [2] |
| pCfB3050 (p-gRNA XII-5) |  |  | [2] |
| pCfB11397 | pTAJAK71 (pESC-NATMX) | BB5651, BB5652 | This study |
| pCfB11398 | pTAJAK71 (pESC-NATMX) | BB5653, BB5654 | This study |
| pCfB11399 | pTAJAK71 (pESC-NATMX) | BB5655, BB5656 | This study |
| pCfB11400 | pTAJAK71 (pESC-NATMX) | BB5657, BB5658 | This study |
| **Integration Plasmids** |  |  |  |
| pCfB9703 | pCfB2899 | BB10, BB4151, BB4440 | This study |
| pCfB10106 (X-3_Ptef1-DbB5GT) | pCfB3035 | BB8, BB4273 | This study |
| pCfB10368 (X-3_UGT73DN1) | pCfB3035 | BB8, BB5051 | This study |
| pCfB10369 (X-3_UGT73A36) | pCfB3035 | BB8, BB5052 | This study |
| pCfB10370 (X-3_UGT73A38) | pCfB3035 | BB8, BB5053 | This study |
| pCfB10371 (X-3_UGT73A39) | pCfB3035 | BB8, BB5054 | This study |
| pCfB11390 (X-2-PgDOD*_full) | pCfB2899 | BB8, BB5638 | This study |
| pCfB11391 (X-2-PgDOD*_truncated) | pCfB2899 | BB8, BB5639 | This study |
| pCfB10954 (XII-5_UGT73A36) | pCfB2909 | BB8, BB5448 | This study |
| pCfB11613 (XII-5_BgDOD1-AnTYH) | pCfB2909 | BB10, BB4697, BB4687 | This study |
| pCfB11789 (pX-2-PgDOD) | pCfB2899 | BB8, BB4688 | This study |
| pCfB11790 (pX-2-BgDOD1) | pCfB2899 | BB8, BB4687 | This study |

Table S5: List of yeast strains constructed in this study

| **Name** | **Parent** | **Added DNA Element(s)** | **Relevant Genotype** | **Source** |
| --- | --- | --- | --- | --- |
| CEN.PK 113-5D |  |  | MATa Δura3 HIS3 LEU2 TRP1 MAL2-8c SUC2 | Peter Kötter |
| BY4741 |  |  | MATa his3Δ0 leu2Δ0 met15Δ0 ura3Δ0 | Euroscarf strain collection |
| ST8251 | CEN.PK 113-5D | pCfB2312 | CEN.PK113-5D *↑Cas9_KanMX* | [3] |
| ST9771 | BY4741 | pCfB2312 | BY4741 *↑Cas9_KanMX* | This study |
| ST10319 | ST8251 | BB0629, BB4773, BB4774, BB4732, BB0630 | CEN.PK113-5D, ↑Cas9, CAN1::(Ptef1-MjDOD-Tcyc1-Ptdh3-BvCYP76AD^W13L^-Tadh1-KlURA3) | This study |
| ST10528 | Lib3-iso2 | pCfB2312 (TEF1p-Cas9-CYC1t_kanMX) | - | This study |
| ST10613 | ST10528 | pCfB10368 (X-3_UGT73DN1) | Iso2 ↑Cas9, Ptef1-UGT73DN1-Tadh1 | This study |
| ST10614 | ST10528 | pCfB10369 (X-3_UGT73A36) | Iso2 ↑Cas9, Ptef1- UGT73A36-Tadh1 | This study |
| ST10615 | ST10528 | pCfB10370 (X-3_UGT73A38) | Iso2 ↑Cas9, Ptef1- UGT73A38-Tadh1 | This study |
| ST10616 | ST10528 | pCfB10371 (X-3_UGT73A39) | Iso2 ↑Cas9, Ptef1- UGT73A39-Tadh1 | This study |
| ST10617 | ST10528 | pCfB10106 (X-3_Ptef1-DbB5GT) | Iso2 ↑Cas9, Ptef1- DbB5GT-Tadh1 | This study |
| ST10780 | ST8251 | BB0629, BB4735, BB4774, BB4732, BB0630 | CEN.PK113-5D, ↑Cas9, CAN1::(Ptef1-PgDOD-Tcyc1-Ptdh3-AnTYH-Tadh1-KlURA3) | This study |
| ST11825 | ST10529 | pCfB10954 (XII-5_Ptef1-UGT73A36) | Lib3-iso21, Ptef1-UGT73A36-Tadh1 | This study |
| ST12267 | ST9771 | pCfB9703 (X-2_*Mj*DOD-TyrHW13L), pCfB10954 (XII-5_Ptef1-UGT73A36) | MATa, his3Δ1 leu2Δo met15Δ0 ura3Δ0, Ptef1-MjDOD-TyrH^W13L^, Ptef1-UGT73A36-Tadh1 | This study |
| ST12269 | ST9771 | pCfB9703 (X-2_*Mj*DOD-TyrHW13L) | MATa, his3Δ1 leu2Δo met15Δ0 ura3Δ0, Ptef1-*Mj*DOD--Tadh1, Ppgk1-TyrH^W13L^-Tcyc1 | This study |
| ST12270 | ST8251 | pCfB9703 (X-2_*Mj*DOD-TyrHW13L) | CEN.PK113-5D *↑Cas9_KanMX,* Ptef1-*Mj*DOD--Tadh1, Ppgk1-TyrH^W13L^-Tcyc1 | This study |
| ST12280 | ST8251 | pCfB11613 (XII-5_BgDOD1-AnTYH) | CEN.PK113-5D *↑Cas9_KanMX,* Ptef1-*Bg*DOD1-Tadh1, Ppgk1-*An*TYH-Tcyc1 | This study |
| ST12383 | ST12267 | pCfB11397 (QDR1_dis) | MATa, his3Δ1 leu2Δo met15Δ0 ura3Δ0, Ptef1-MjDOD-TyrHW13L, Ptef1-UGT73A36, QDR1_dis | This study |
| St12384 | ST12267 | pCfB11398 (QDR2_dis) | MATa, his3Δ1 leu2Δo met15Δ0 ura3Δ0, Ptef1-MjDOD-TyrHW13L, Ptef1-UGT73A36, QDR2_dis | This study |
| ST12385 | ST12267 | pCfB11399 (ALP1_dis) | MATa, his3Δ1 leu2Δo met15Δ0 ura3Δ0, Ptef1-MjDOD-TyrHW13L, Ptef1-UGT73A36, ALP1_dis | This study |
| ST12386 | ST12267 | pCfB11400 (YJR015W_dis) | MATa, his3Δ1 leu2Δo met15Δ0 ura3Δ0, Ptef1-MjDOD-TyrHW13L, Ptef1-UGT73A36, YJR015W_dis | This study |
| ST12515 | ST12280 | pCfB11789 (X-2-PgDOD) | CEN.PK113-5D *↑Cas9_KanMX,* Ptef1-*Bg*DOD1-Tadh1, Ppgk1-*An*TYH-Tcyc1, Ptef1-*Pg*DOD-Tadh1 | This study |
| ST12516 | ST12280 | pCfB11790 (X-2-BgDOD1) | CEN.PK113-5D *↑Cas9_KanMX,* Ptef1-*Bg*DOD1-Tadh1, Ppgk1-*An*TYH-Tcyc1, Ptef1-*Bg*DOD1-Tadh1 | This study |
| ST12517 | ST12280 | pCfB11390 (X-2-PgDOD*_full) | CEN.PK113-5D *↑Cas9_KanMX,* Ptef1-*Bg*DOD1-Tadh1, Ppgk1-*An*TYH-Tcyc1, Ptef1-*Pg*DOD*-Tadh1 | This study |
| ST12518 | ST12280 | pCfB11391 (X-2-PgDOD*_truncated) | CEN.PK113-5D *↑Cas9_KanMX,* Ptef1-*Bg*DOD1-Tadh1, Ppgk1-*An*TYH-Tcyc1, Ptef1-tr*Pg*DOD*-Tadh1 | This study |
| ST12379 | OneShot BL21 (DE3) | pCfB11487 (pET-BvSGT2) | BL21(DE3): F- ompT hsdSB (rBmB-) gal dcm (DE3) *↑PT7-*6xHis-BvSGT2 | This study |


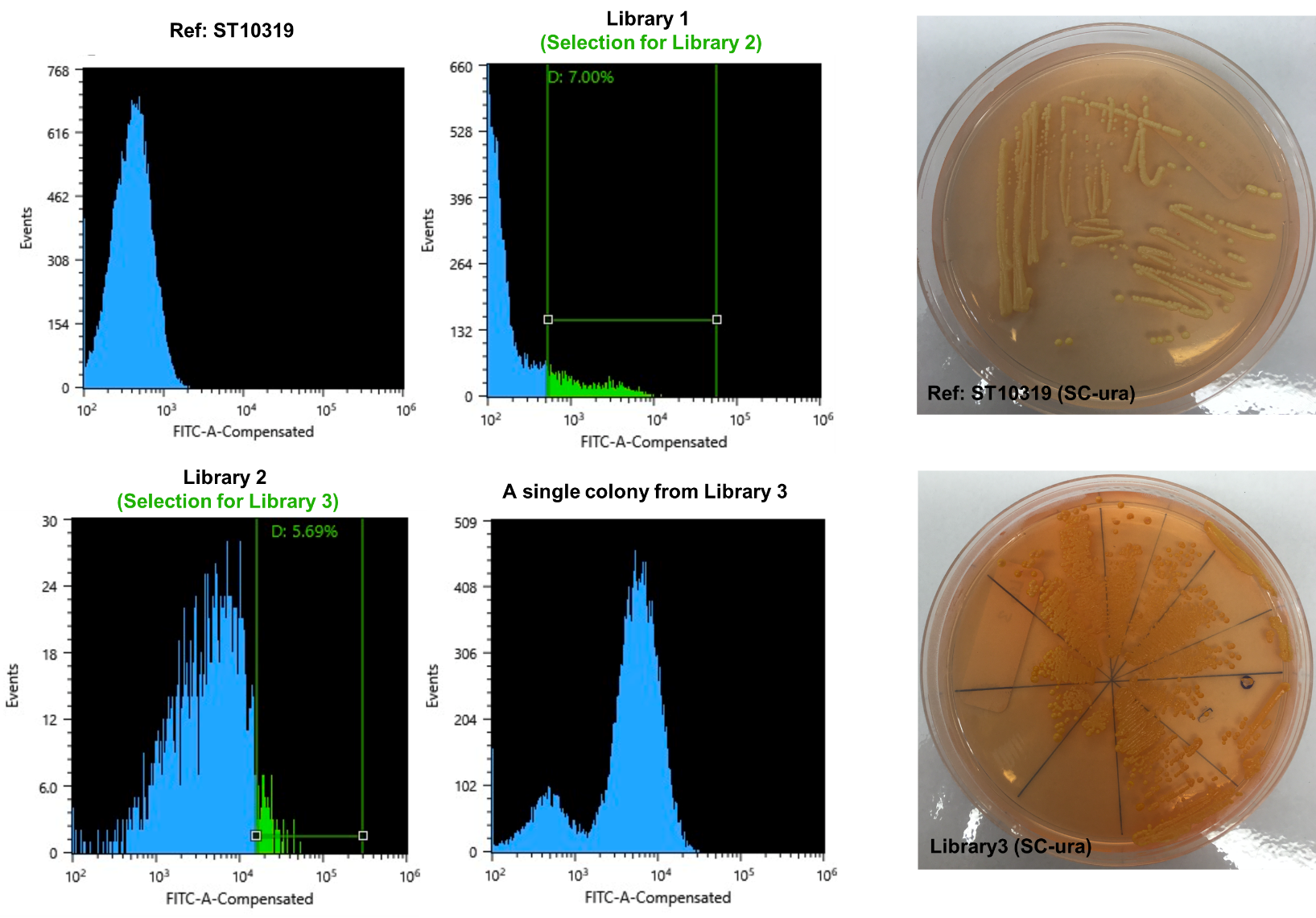


Figure S1: The fluorescence of cell populations used for FACS, and also the cell color difference for ST10319 and Library3 on SC-ura plates


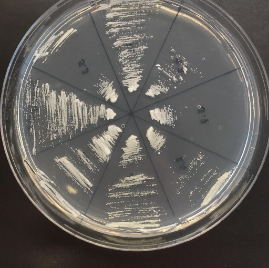


Parent

*Pg*DOD^T732∆^ -*An*TyH

*Pg*DOD^T732∆^ -*Ev*TyH

Figure S2: Cultivation of multiple colonies obtained by integrating *Pg*DOD^T732∆^ together with two different TyH genes into genome of *S. cerevisiae*. The media is minimal media without pABA. The parent strain is CEN.PK113-7D. The strains were constructed using EasyClone toolbox, and the correct integration of fragments into genome was verified by colony PCR.


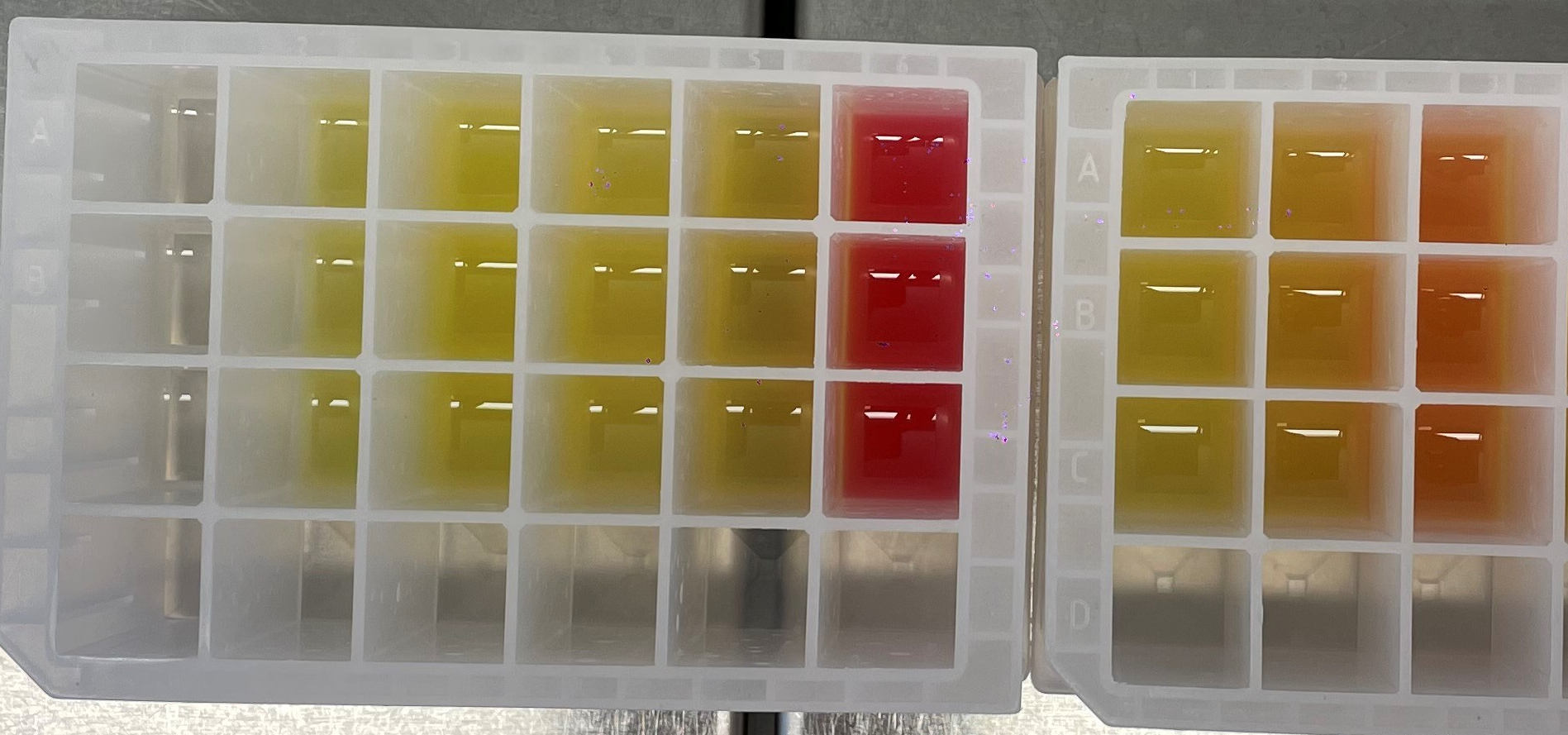


**Parent iso2 iso16 iso21 UGT73DN1 UGT73A36 UGT73A38 UGT73A39 DbB5GT**

Figure S3: Betalain production in yeast strains. The photos are taken after 48 hours of growth in MM (pABA^-^). The parent strain for integration of glucosyltransferases is iso2.

Figure S4: MATGAT analysis for DOD enzyme variants


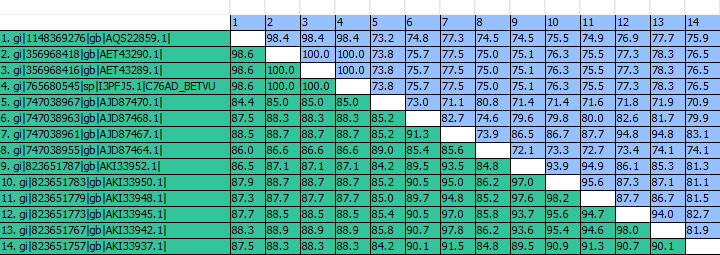


Figure S5: MATGAT analysis for CYP76ADα (termed as TYH in this study) enzyme variants


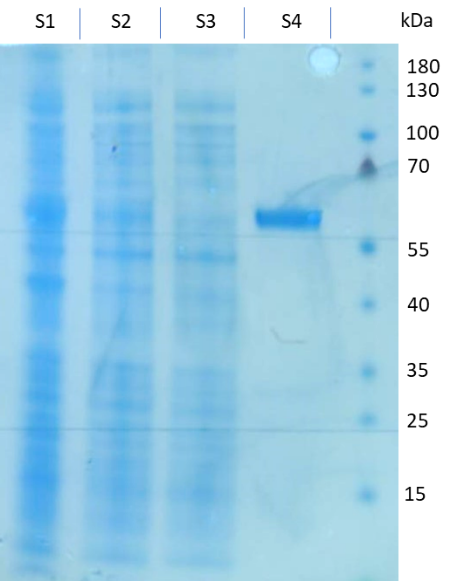


Figure S6: SDS-PAGE of fractions collected during purification of recombinantly expressed UGT73A36. S1: lysed cells after sonification, S2: soluble protein fraction after cell lysis, centrifugation and filtration, S3: Flow-through after sample loading on the HiTrap column, S4: Pooled and up-concentrated elution fractions. Samples were mixed 1:1 with sample buffer (+DTT), heated at 95 °C for 10 min, separated via SDS-PAGE and stained with InstantBlue Coomassie Protein Stain.


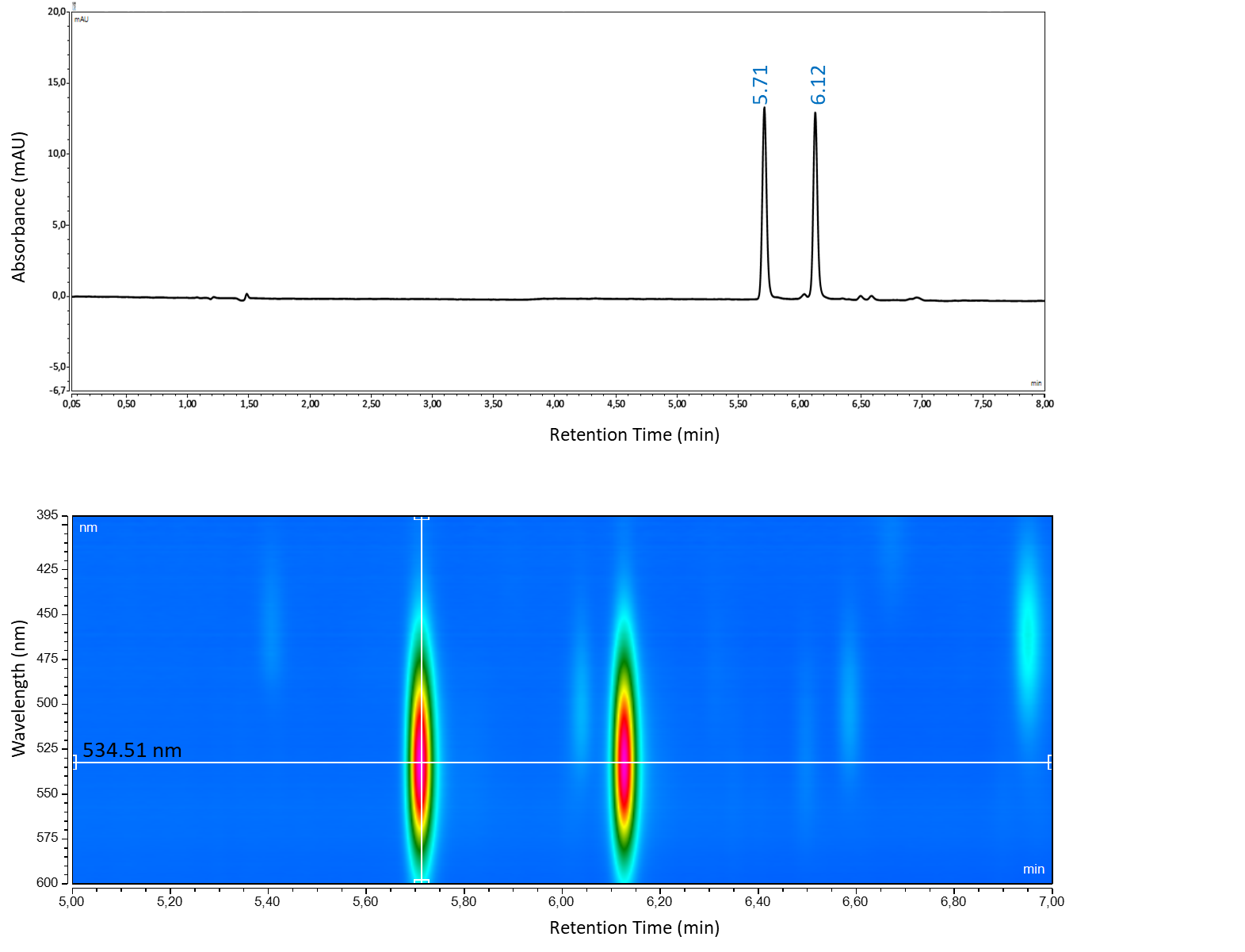


Figure S7: HPLC-chromatogram and 3-D contour plot of betanin standard solution (1 g/L) from Sigma-Aldrich. The peaks at 5.71 and 6.12 min correspond to betanin and isobetanin, respectively.


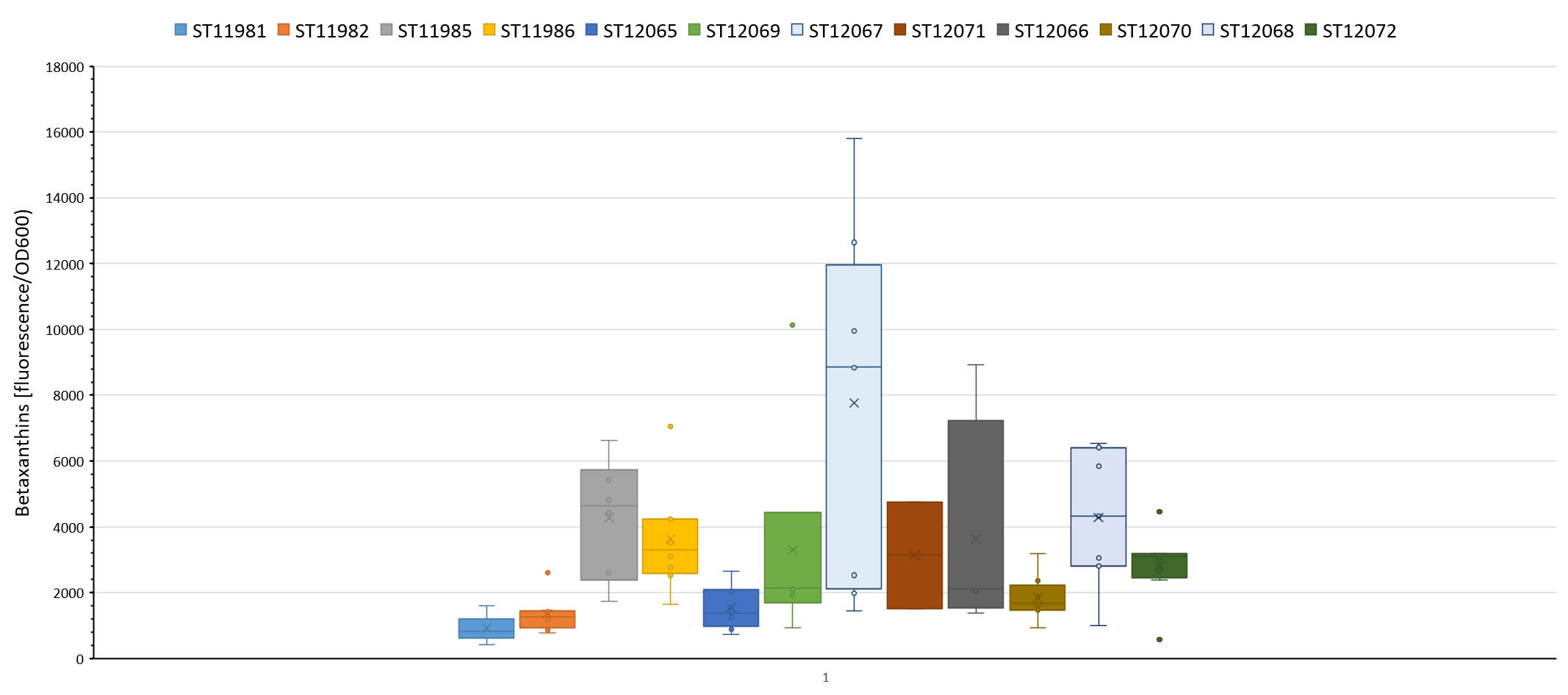

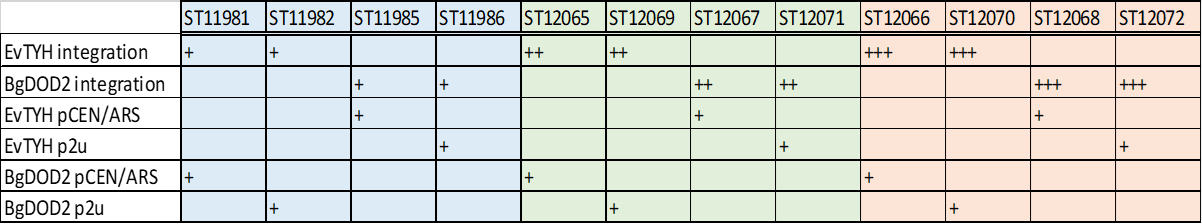

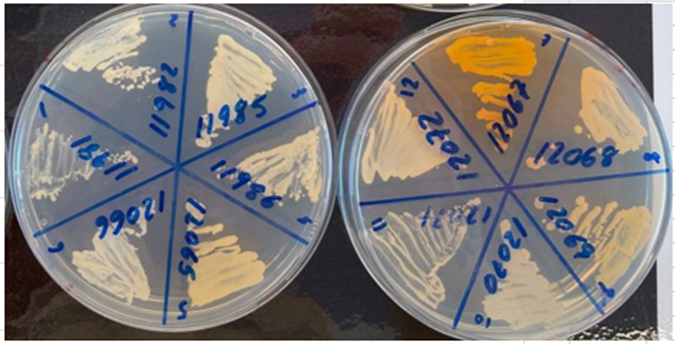


Figure S8: Effect of TYH-DOD expression ratio on betaxanthins titer.

**References**

1. Stovicek V, Borodina I, Forster J. CRISPR–Cas system enables fast and simple genome editing of industrial Saccharomyces cerevisiae strains. Metabolic Engineering Communications. 2015 Dec;2:13–22.

2. Jessop-Fabre MM, Jakočiūnas T, Stovicek V, Dai Z, Jensen MK, Keasling JD, et al. EasyClone-MarkerFree: A vector toolkit for marker-less integration of genes into Saccharomyces cerevisiae via CRISPR-Cas9. Biotechnology Journal. 2016;

3. van der Hoek SA, Darbani B, Zugaj KE, Prabhala BK, Biron MB, Randelovic M, et al. Engineering the Yeast Saccharomyces cerevisiae for the Production of L-(+)-Ergothioneine. Frontiers in Bioengineering and Biotechnology. 2019 Oct 11;7.
